# All-optical control of nonlinear emission from resonant metasurfaces

**DOI:** 10.1126/sciadv.aeh0904

**Published:** 2026-09-25

**Authors:** Ziwei Yang, Lei Xu, Gabriel Sanderson, Akhshay Bhadwal, Luyao Wang, Katsuya Tanaka, Muyi Yang, Mingkai Liu, Shaun Lung, Isabelle Staude, Thomas Pertsch, Carl Brown, Mohsen Rahmani, Dragomir Neshev

**Affiliations:** ^1^ ARC Centre of Excellence for Transformative Meta-Optical Systems (TMOS), Department of Electronics Materials Engineering, Research School of Physics, The Australian National University, ACT 2601, Australia.; ^2^ Institute of Applied Physics, Abbe Center of Photonics, Friedrich Schiller University Jena, Jena 07745, Germany.; ^3^ Advanced Optics and Photonics Laboratory, Department of Engineering, School of Science and Technology, Nottingham Trent University, Nottingham NG11 8NS, UK.; ^4^ SOFT Group, Department of Physics and Mathematics, School of Science and Technology, Nottingham Trent University, Nottingham NG11 8NS, UK.; ^5^ Institute of Solid State Physics, Friedrich Schiller University Jena, Jena 07745, Germany.

## Abstract

Nonlinear optics underpins a broad range of photonic technologies, from classical and quantum light sources to emerging nonlinear photonic neural networks. Yet, conventional nonlinear-optical devices exhibit static functionality: Their transfer characteristics and emission profiles are dictated by the intrinsic nonlinear process and locked by fabrication, limiting adaptability. Here, we introduce an ultrathin metasurface platform that enables dynamic reconfiguration of nonlinear functionality in a contactless fashion. By leveraging all-optical control of the optical torque exerted on liquid crystal molecules infiltrating a resonant metasurface, we achieve tunable polynomial nonlinear transfer functions based on the third-harmonic generation process. This mechanism further allows reconfigurable modulation of nonlinear weighting across different diffraction orders, revealing a previously unexplored interplay between mode structure and nonlinear emission. Our approach opens up a pathway toward field-programmable nonlinear photonic systems, offering unprecedented flexibility for reconfigurable nonlinear signal processing and adaptive photonic computing.

## INTRODUCTION

Nonlinear optical processes (*1*) form a cornerstone of contemporary optics, underpinning key functionalities such as frequency conversion (*2*, *3*), optical signal processing (*4*), and nonlinear wave-matter interactions (*5*) being used in cutting-edge technologies such as lasers, optical fibers, and high-precision biochemical imaging (*6*, *7*). Efficient nonlinear interactions typically require strong light confinement or long interaction lengths, which are satisfied with relatively large nonlinear crystals in today’s macroscale devices. However, the high cost of nonlinear crystals and the limited space in miniaturized devices have driven a scientific quest to generate and control nonlinear emissions with minimal reliance on material volume. Such a need has given birth to nonlinear photonics, motivating the development of resonant nanophotonic structures that enhance electromagnetic fields at subwavelength scales. In this context, metasurfaces have emerged as a compact and versatile platform for nonlinear optics, offering precise control over resonance properties, field distributions, and radiation channels.

The subwavelength resonances supported by metasurfaces lead to strong electromagnetic field confinement, which enhances light-matter interactions and enables efficient nonlinear processes such as harmonic generation (*8*–*15*) and frequency mixing (*16*–*18*). This resonant enhancement enables efficient nonlinear emission from ultrathin devices and allows tailoring of spectral, spatial, and polarization responses. Furthermore, the same resonant nature that enables strong nonlinear enhancement also renders metasurfaces highly sensitive to external perturbations, providing a natural pathway toward dynamically reconfigurable nonlinear functionality (*19*–*21*).

Actively tunable metasurfaces (*22*, *23*) have therefore emerged as a central platform for next-generation flat optics, offering unprecedented dynamic control over light-matter interactions at subwavelength scales. Unlike static devices whose functionality is fixed after fabrication, active metasurfaces enable real-time modulation of optical responses and have been widely explored for dynamic beam steering (*24*–*27*), focusing (*28*, *29*), programmable holography (*30*, *31*), and optical information processing (*32*–*34*). However, the effectiveness of a tunable metasurface ultimately depends on the availability of robust mechanisms that enable reversible, high-speed, and low-loss modulation of its optical response.

The tunability of metasurfaces originates from external stimuli that modify the material refractive index (*35*–*37*), the ambient environment (*38*, *39*), or the structural geometry (*40*–*42*). Such perturbations alter the resonance conditions of the metasurface and consequently modulate its optical responses. Owing to the strong electromagnetic field confinement within metasurfaces, even small variations in these parameters can lead to substantial changes in resonance frequency, linewidth, and field distribution. This sensitivity enables diverse tuning mechanisms based on electrical (*43*–*47*), thermal (*48*–*50*), or optical excitation (*35*, *51*–*53*). When applied to nonlinear metasurfaces, these tuning mechanisms provide a means to dynamically control nonlinear emission intensity, spectral position, and efficiency through the modulation of resonant field enhancement.

Despite these advances, the dynamic modulation of nonlinear metasurfaces remains a formidable challenge. Conventional tuning approaches, including thermal, electrical, and mechanical actuation, suffer from intrinsic limitations—thermal methods are slow, electrical schemes require electrodes that may introduce losses or fabrication complexity, and mechanical modulation lacks scalability. More fundamentally, although spectral resonance shifts can modulate nonlinear conversion efficiency, such tuning does not fundamentally reconfigure the spatial field profile or mode overlap that governs nonlinear source generation. As a result, conventional approaches primarily enable amplitude modulation rather than true functional reconfiguration of nonlinear pathways. Achieving dynamic control over nonlinear functionality, therefore, requires in situ reshaping of the local electromagnetic field distribution and multipolar interactions. Identifying a contactless and reversible tuning mechanism capable of directly modifying the modal environment, while simultaneously influencing both linear resonances and nonlinear field enhancement, is therefore of critical fundamental importance.

Liquid crystals (LCs) provide a uniquely versatile solution to this challenge. Their long-range molecular order provides large and continuously adjustable birefringence, enabling smooth modulation of the refractive index across visible and near-infrared wavelengths. Owing to their fluidic nature, LCs can conformally surround nanostructures and efficiently interact with the confined near fields of metasurfaces. As a result, small orientation-induced changes in the LC director can produce pronounced modifications of the resonant response. This intimate coupling makes LCs highly compatible with resonant metasurfaces, enabling effective tuning of both linear (*54*–*56*) and nonlinear (*20*) optical responses. However, the tunability of metasurface-LC systems generally depends on external control mechanisms, such as wire-connected electrodes for electrical biasing, localized thermal heating, or chemical engineering of prealignment layers to enforce director reorientation. These approaches introduce structural complexity and practical constraints that limit the full reconfigurable potential of LCs in hybrid photonic platforms.

In this work, we use an optical torque–driven tuning mechanism that realizes purely optical reconfiguration of metasurface-LC systems, in a contactless fashion. By encapsulating a dielectric metasurface within a Teflon-aligned LC cell, we exploit the reorientation of LC molecules induced by the polarization-dependent optical torque of the excitation beam. This mechanism provides dynamic reversible tuning of the local refractive index up to Δn = 0.2 anisotropy without requiring heating or electrodes. Here, the term dynamic tuning indicates reversible optical modulation between quasi-static LC metasurface states, achieved on a millisecond time frame rather than on the femtosecond scale of the laser pulse. Using this purely optical stimulus not only enables us to modulate the linear optical properties of the system but also provides the opportunity for a unique nonlinear polynomial modulation, i.e. third-harmonic generation (THG) in our case, arising from optical torque–induced tuning of the resonant mode. It modifies the local field enhancement and, consequently, the effective nonlinearity of the system. Furthermore, we observe power redistribution among diffraction orders in the nonlinear regime, establishing a link between mode symmetry, nonlinear emission, and far-field beam shaping.

This study establishes a theoretical and experimental paradigm for all-optical control of nonlinear metasurfaces. Fundamentally, it reveals a higher-order relationship between resonant modulation and effective nonlinear susceptibility, demonstrating that external optical excitation can directly govern the strength and symmetry of nonlinear emission. Moreover, the developed framework introduces an external force–driven nonlinear temporal coupled-mode theory, which quantitatively describes the dynamic interplay between optical torque, resonant mode evolution, and harmonic generation. By controlling nonlinear dynamics via the optical torque, this work bridges the gap between microscopic reorientation processes and macroscopic field modulation. Such a framework not only advances the theoretical understanding of tunable nonlinear photonics but also enables practical implementations of contactless and reversible spatial light modulation, optically driven multifunctional meta-devices, neuromorphic photonic architectures, and adaptive nonlinear imaging.

## RESULTS

### Conceptual demonstration of the tuning mechanism

**Fig. 1.**
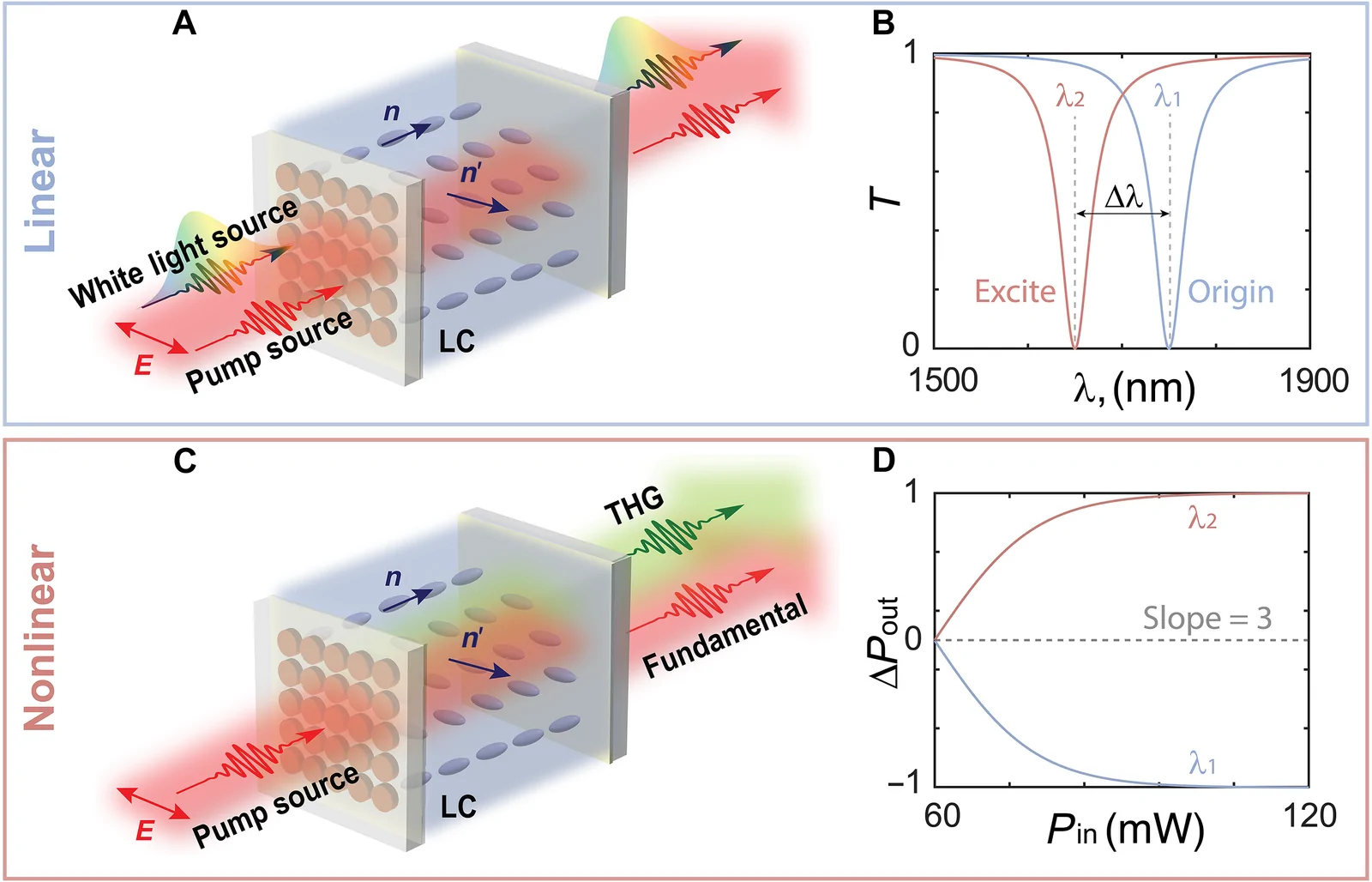
Conceptualized illustration of linear and nonlinear optical response in LC tunable metasurfaces. (**A**) Schematic of linear response influenced by LC rotation driven by polarization-induced optical torque. (**B**) Transmittance spectrum of resonance shift due to LC rotation. (**C**) Demonstration of the THG from a silicon metasurface controlled by LC rotation. (**D**) Positive and negative deviations of the third-harmonic output caused by the resonance shift.

The conceptual illustration underlying contactless, reversible, and broadband light manipulation via optical torque is presented in Fig. 1. As can be seen in Fig. 1A, our system uses LC as an anisotropic medium for all-optical tuning. By adjusting the orientation of the nematic LC director, one can effectively change its refractive index tensor. In contrast to conventional tuning, here, the LC director is oriented by the optical torque induced by linearly polarized light, referred to as polarization-induced optical torque (PIOT) (*57*). Since the LC response is much slower than the femtosecond excitation, the director follows the time-averaged PIOT of the pulse train, with the average pump power setting the reorientation strength (*58*, *59*). By integrating this optically driven LC environment with dielectric meta-resonators that support electric and magnetic multipolar modes, the PIOT-induced index change can be translated into resonance tuning in both the linear and nonlinear regimes.

In this configuration, the LC infiltrates the space surrounding the silicon meta-resonators and is initially homeotropically aligned, with the director perpendicular to the metasurface plane (*60*). A spectrally detuned near-infrared femtosecond pump applies optical torque to the LC and reorients the director toward the metasurface plane, while a broadband white-light source probes the linear optical response. This pump-induced reorientation modifies the anisotropic environment surrounding the resonators and shifts the metasurface resonance from its initial state, as illustrated in Fig. 1B.

To implement PIOT in the LC-metasurface system, we first prepare a homeotropic alignment layer to prealign the LC director perpendicular to the metasurface plane. Upon the application of an external force, specifically an electric field by the pump, the LC director undergoes reorientation toward a direction parallel to the metasurface plane. This reorientation modifies the anisotropic environment surrounding the metasurface, thereby altering its optical response. By characterizing the far-field optical transmission spectra, we observe a clear resonance shift from the unexcited resonance state of the metasurface driven by the LC reorientation due to PIOT, as illustrated in Fig. 1B.

Beyond the linear response, the same PIOT mechanism also provides a unique modulation opportunity in the nonlinear regime. It enables pronounced modulation of the nonlinear response, which, to our knowledge, has not been reported in other nonlinear metasurface systems to date. As illustrated in Fig. 1C, an infrared pump acts both as the LC-driving field and as the excitation source for the nonlinear process. The LC director responds to the time-averaged optical torque of the pulse train on a millisecond timescale and is therefore quasi-static with respect to the femtosecond-driven THG process, providing a slowly varying resonant environment for nonlinear emission. When this pulse excites the centrosymmetric Si-based metasurface, THG is generated from the metasurface. Because the LC orientation depends on the driving pump field intensity, increasing the pump power changes not only the nonlinear source strength but also the LC-mediated resonance detuning. This coupling between nonlinear excitation and resonance tuning modifies the THG response beyond the conventional cubic power dependence, enabling an actively tunable platform that further enriches the overall nonlinear responses based on the resonant excitation and interference effects.

When the fixed pump wavelength $\lambda_{1}$ is initially aligned with the metasurface resonance, increasing the pump power gradually shifts the resonance away from $\lambda_{1}$, leading to progressive pump-resonance detuning. By contrast, when the pump wavelength is fixed at $\lambda_{2}$, increasing the pump energy progressively shifts the resonance toward $\lambda_{2}$, enhancing the pump-resonance overlap. This dynamic spectral tuning gives rise to a polynomial nonlinear transfer function governed by the evolving resonance detuning. Instead of the conventional cubic THG relation with a slope of 3 on a logarithmic plot, the nonlinear response exhibits opposite deviations at $\lambda_{1}$ and $\lambda_{2}$, as shown in Fig. 1D, Under conventional conditions, the TH energy follows a cubic relation with input pump power, yielding a straight line with a slope of 3 on a logarithmic plot. However, when incorporating the dynamic detuning effect on a logarithmic scale, the nonlinear response exhibits a pair of opposing deviations in slope at $\lambda_{1}$ and $\lambda_{2}$ (with a straight slope indicating no deviation), as shown in Fig. 1D, where $\Delta P_{\text{out}}$ denotes the deviation of the output power from that obtained without dynamic detuning. This effect reflects a gradual, higher-order nonlinear modulation superimposed on the conventional THG process.

### PIOT in LC-infiltrated metasurfaces

**Fig. 2.**
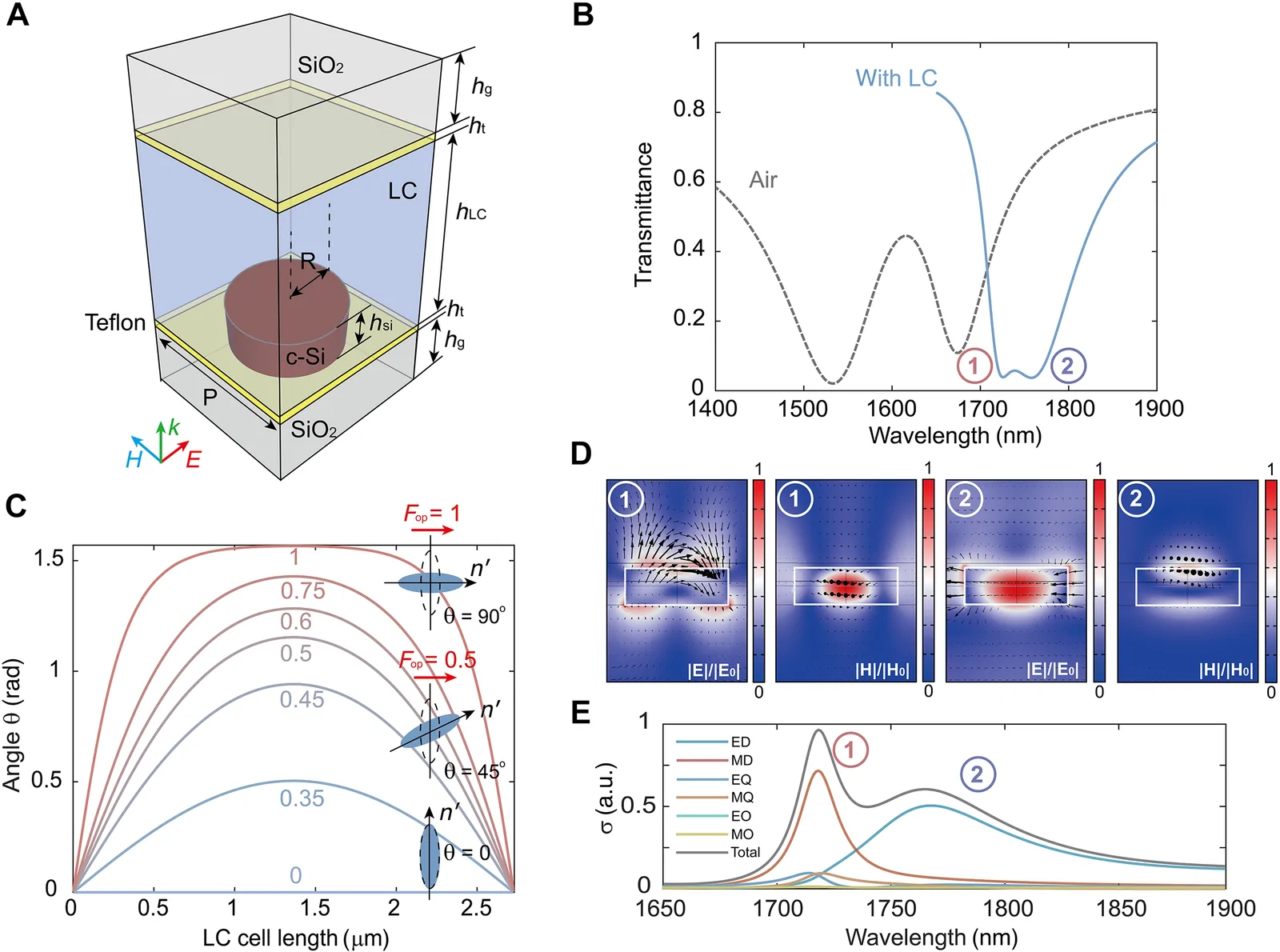
Theoretical optical-response of the silicon-based metasurface infiltrated with E7 LC. (**A**) Three-dimensional view of the metasurface unit cell, the prealignment material Teflon is coated on both sides of the LC cell to provide a hard-boundary condition for homeotropic alignment. By giving the light excitation from the back of the metasurface, we designed parameters of the unit cell as P=955 nm, $h_{t}=150$ nm, $h_{\text{Si}}=245$ nm, $h_{\text{LC}}=2.85\;\mu m$, and R=350 nm, and $h_{g}$ is infinity. (**B**) The simulated transmittance of the designed metasurface before and after LC infiltration. Two modes are labeled by number 1 (light red) and 2 (light purple). Calculated LC space-inhomogeneous rotation angles with different normalized electric fields are shown in (**C**). Here, the x axis represents the height of the LC cell, while the y axis indicates the LC rotation angle in the *xz* plane, in radians. Maximum LC rotation angle in radians is 1.57 ($\approx 90^{\circ }$). (**D**) Normalized magnitudes of electric and magnetic fields for two modes with LC infiltration. Field analysis using multipolar decomposition in (**E**) shows that mode 1 (resonance 1) is the magnetic dipole dominant and mode 2 (resonance 2) is the electric dipole dominant.

To gain an in-depth insight into the physics process in a realistic scenario, we first design a crystalline silicon metasurface on a quartz substrate, as is shown in Fig. 2A. The simulated transmittance in air, obtained under normal incidence, is shown by the gray dashed curve in Fig. 2B, revealing two fundamental resonances at 1536 and 1676 nm. A Teflon layer is subsequently introduced as a prealignment layer, which imposes a homeotropic initial director orientation perpendicular to the metasurface plane. In addition, an LC (E7) layer is then included as the surrounding medium, together with a semi-infinite top cover layer coated with an identical Teflon layer, with the same thickness as the bottom one. After incorporating all parameters into the model, the resulting LC-infiltrated metasurface transmittance is represented by the blue curve, where the two resonances become spectrally overlapped because of the surrounding refractive-index change.

To model the dynamic behavior of the PIOT-driven LC metasurface in full three dimensions under varying pump powers, we calculate the LC director profile using a continuum orientation model. We assume that the LC molecules strongly anchored near the metasurface surface and remain fixed, while the LC region above the metasurface can be reoriented by the optical torque. This approximation allows the director rotation to be described by the optical Freedericksz transition (*57*) (detailed equation derivations see section S1)

$$K\frac{d^{2}\theta }{{\mathrm{dz}}^{2}}+\frac{\varepsilon_{0}\Delta \varepsilon }{4}\text{sin}2\theta {|E_{x}|}^{2}=0$$ (1)

Here, the K denotes the elastic constant of LC, and $\Delta \varepsilon =\varepsilon_{∥}-\varepsilon_{⊥}=0.72$ for E7, where the $\varepsilon_{∥}$ and $\varepsilon_{⊥}$ are the optical dielectric constants parallel and perpendicular to the LC director, respectively.

To reorient the LC molecules via the PIOT, the applied optical torque must be much greater than the elastic torque. In Eq. 1, the $\frac{\varepsilon_{0}\Delta \varepsilon }{4}{|E_{x}|}^{2}$ term represents the effective optical torque acting on the LC under x-polarized light. We also define the bottom surface of the silicon cylinder as the origin in Cartesian coordinates, applying the boundary conditions $\theta \left(h_{\text{Si}}\right)=\theta \left(h_{\text{LC}}\right)=0$. The resulting LC rotation profiles as a function of distance are shown in Fig. 2C, where the continuous LC rotation angles are calculated according to different pump power levels with coded colors, illustrating a smooth, continuous rotation along the light propagation direction. The inset LC molecules illustrate the relationship between the LC director orientation and the normalized optical dipolar interaction energy $F_{\text{op}}$. To benchmark the required power level, we further provide an order-of-magnitude estimate of the incident intensity for optically driven reorientation based on a small-angle torque balance, $I_{\text{th}}\approx \mathrm{ncK}/\Delta \varepsilon h_{\text{LC}}^{2}\approx$ 0.896 kW/cm^2^, which is consistent with the experimental pumping conditions (see section S1 for details).

It is worth noting that the total LC thickness is chosen to be 3 μm, as our simulations confirm that the metasurface near-field scattering response is negligibly affected by refractive index variations occurring above 1 μm. Using the calculated LC director angle distribution, we derive the effective anisotropic refractive index by applying a rotation matrix through Euler angle transformation. The general description of crystalline axes rotation is shown as Eq. 2

$$\hat{n}=\hat{\rho }\left(\alpha ,\theta \right)\;\left[\begin{array}{ccc} n_{o} & 0 & 0\\ 0 & n_{o} & 0\\ 0 & 0 & n_{e} \end{array}\right]\;\hat{\rho }{\left(\alpha ,\theta \right)}^{-1}$$ (2)

In Eq. 2, $\hat{\rho }$ is the rotation matrix, α represents the rotated angle in the *xy* plane, and θ denotes the angle between the LC director and the z axis (see section S1 for details).

Building on the anisotropic refractive index distribution derived from the LC rotation profile, we further investigate how these spatial anisotropies affect the mode response. As we discussed in previous work (*38*), the two resonances exhibit different optical responses because their field distributions sample different regions of the LC-infiltrated metasurface. As plotted in the normalized electric and magnetic fields in Fig. 2D, the modal fields extend into the surrounding E7 LC rather than being fully confined inside the Si cylinders. To rigorously classify the resonances, we perform a multipolar decomposition analysis. The results in Fig. 2E indicate that the resonance labeled as 1 (red curve) is a magnetic dipole–dominated mode, whereas resonance 2 (blue curve) exhibits a dominant electric dipole character.

### Linear experimental characterization

**Fig. 3.**
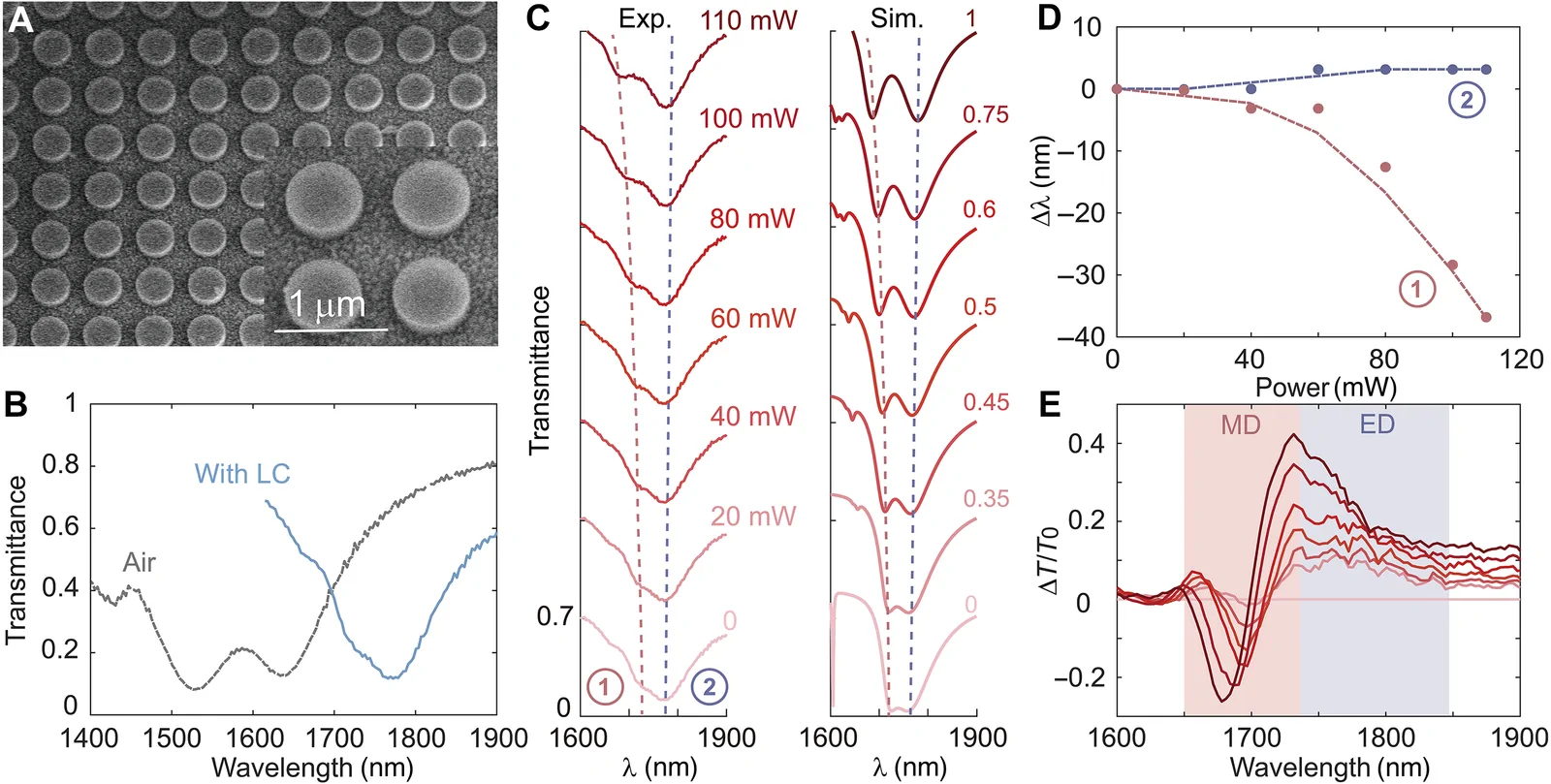
Fabrication and experimental characterization of the linear optical-response. (**A**) SEM images showing a bare metasurface coated with a thin Teflon layer, where the Teflon molecules are uniformly distributed around the nanodisk array. (**B**) The measured transmittance of the metasurface before and after LC infiltration. Comparing the transmittance results from both experiment and simulation in (**C**), the resonances 1 and 2 illustrate good consistency as the laser average power increases. By extracting the experimental data along the red and blue dashed lines in (C), we track the shifts of the two resonances, as shown in (**D**), where the dots come from the experiment, and the two dashed lines are tracked from the simulation. In addition, the power-dependent modulation of transmittance under resonant wavelength is presented in (**E**), which shows the ratio of transmittance difference at the initial (1716 nm) and final (1685 nm) resonance position.

Guided by our simulations, we fabricated a Si metasurface using standard electron-beam lithography and etching processes. Following fabrication, a Teflon layer was coated onto the structure, as shown in the scanning electron microscopy (SEM) image in Fig. 3A. Details of the coating process are provided in section S2. In this SEM image, the rough background corresponds to the Teflon layer, which is uniformly coated on the quartz substrate and exhibits nanometer-scale particulates.

Before assembling the LC cell, we characterized the transmittance of the metasurface, labeled “Air,” in Fig. 3B, which agrees well with the simulated static response. We then assembled the LC cell and infiltrated it with E7, following the procedures described in (*38*, *55*). Once the LC molecules were fully reoriented by the prealignment layer, cross-polarized optical microscopy confirmed uniform homeotropic alignment of the LC molecules within the cell (see section S2).

After infiltration, the transmittance spectrum, shown as the light blue curve in Fig. 3B, was measured under normally incident, linearly polarized light (polarization aligned along the metasurface *x* axis). The measured response matches well with the simulation predictions (see the experimental setup in section S3). In this configuration, the magnetic and electric dipole resonances partially overlap, consistent with the simulated results, although the measured spectrum exhibits increased intrinsic losses. Following this static characterization, we proceed to investigate the dynamic modulation of the optical response induced by PIOT.

To enable the modulation by PIOT, we use a spectrally detuned narrowband pulse laser centered at 1450 nm, chosen to reduce resonant absorption and heating, with a pulse duration of 150 fs and a repetition rate of 80 MHz, as the pump beam. The broadband white light source used in the static measurements now serves as the probe beam. The pump beam is spatially expanded to uniformly illuminate the transmittance measurement area and drive LC reorientation. Starting from the static state (labeled as 0) and gradually increasing the average pump power up to 110 mW, we record the transmittance spectra shown in Fig. 3C. As the power increases, the magnetic dipole–dominated resonance (mode 1) exhibits a pronounced blue shift, whereas the electric dipole resonance (mode 2) undergoes only a slight red shift. This trend is consistent with the simulated transmittance spectra at different normalized LC rotation stages, shown on the right-hand side of Fig. 3C, with the numbered labels corresponding to the LC orientations in Fig. 2C.

We attribute the observed resonance shifts to the distinct field distributions of the two modes. Mode 1 is primarily sensitive to the z-component of the refractive index, which decreases from 1.7 to 1.5 during LC reorientation. Its field distribution extends above and below the resonators, allowing it to strongly interact with the modulated refractive index environment. In contrast, mode 2 is sensitive to the x-index component, which increases from 1.5 to 1.7. However, its field is predominantly confined inside and beside the pillars, where the refractive-index change is limited by the static Si and Teflon regions. By tracking the local minima of the resonances in Fig. 3C, we extract a pronounced shift of (Δλ≈36 nm) for mode 1 and a minor shift of ∼3 nm for mode 2, as shown in Fig. 3D. To further confirm that the observed resonance shifts are driven by PIOT rather than thermal accumulation, we performed control measurements using a probe beam with perpendicular polarization. Since a thermal effect would shift the resonances irrespective of probe polarization, the absence of comparable spectral shifts in this configuration supports the conclusion that the modulation arises from PIOT (see section S4 for details).

Next, we plot the corresponding transmittance modulation ratio with different power, $\Delta T/T_{0}$, shown in Fig. 3E. The modulation becomes evident above an incident power of 60 mW, corresponding to an experimental threshold for LC reorientation in our cell to be ∼0.937 kW/cm^2^ in terms of average intensity, and a pulse fluence of ∼11.7 μJ/cm^2^. This threshold indicates that observable PIOT-induced optical modulation occurs only when the PIOT exceeds the elastic force of the LC medium. At 1685 nm, the transmittance decreases relative to the initial state, whereas at 1716 nm it increases, reflecting the separation of the two resonances under optical torque. Therefore, these results confirm PIOT-driven dynamic resonance tuning in the linear regime, when applied optical torque exceeds the intrinsic elastic force.

### THG nonlinear experimental characterization

In the nonlinear optics regime, the pump pulses both drive LC reorientation and excite THG from the metasurface. We therefore investigate the dynamic nonlinear light-matter interaction in a rotating LC nanostructure under high-power laser excitation. Using the same pulse laser as in the linear measurements, we set the pump wavelength at the fundamental resonance regime. The white-light probe is removed from the system, and the generated THG signal is recorded by a spectrometer after short-pass filtering to suppress the residual pump (see section S3 for details).

In our design, the femtosecond laser first interacts with the backside of the metasurface before propagating through the LC domain. This configuration minimizes beam distortion from LC inhomogeneity, which could otherwise destabilize the incident light and interfere with the nonlinear response. Increasing the power of the incident light simultaneously drives LC reorientation and shifts the resonance, leading to dynamic tuning of the nonlinear efficiency relative to a static metasurface with a fixed resonance. We also verified the origin of the nonlinear signal by performing control measurements in regions without the Si metasurface. In these areas, THG was only observed when the pump power exceeded ∼300 mW, indicating that the metasurface plays a dominant role in the THG.

**Fig. 4.**
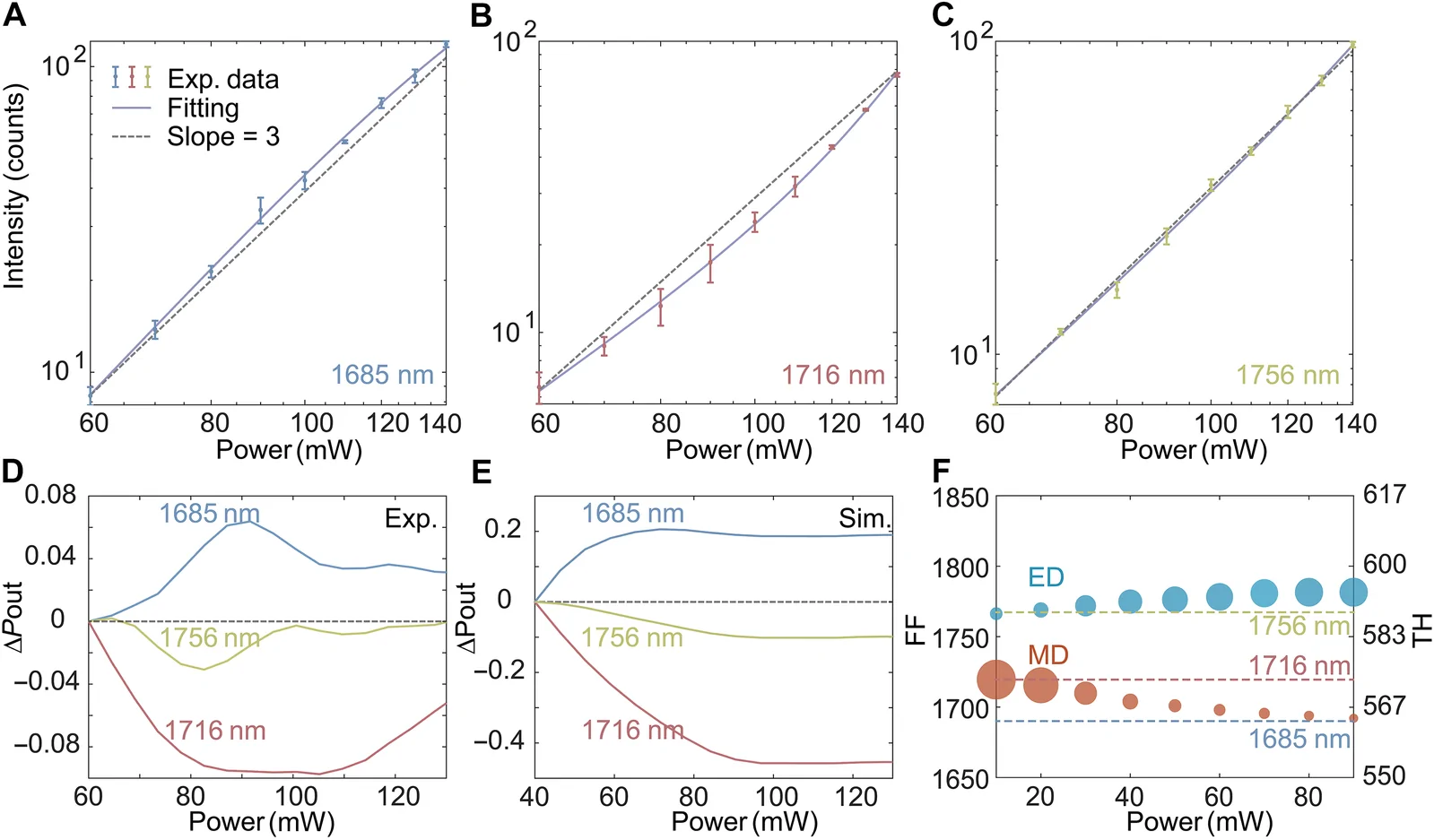
Third-harmonic nonlinear optical-response characterization. Log-log–scaling power-dependent THG plot for the fundamental wavelength (FW) at 1685 nm (blue), 1716 nm (red), and 1756 nm (yellow), corresponding to (**A**) to (**C**), respectively. Experimental (**D**) and simulated (**E**) cubic bending deviations with three picked [(A) to (C)] wavelengths. (**F**) Influence of fundamental resonance shift with respect to THG efficiency according to multipolar decomposition analysis. Here, FF and TH denote the fundamental and third-harmonic frequencies, respectively.

We set the driving source at wavelengths of 1685, 1716, and 1756 nm, corresponding to three representative resonance conditions. At 1685 nm, the magnetic dipole resonance shifts toward the pump wavelength; and at 1716 nm, it shifts away from the pump; and at 1756 nm, the nonlinear generation is attributed to a nearly static electric dipole mode. The measured intensities from the spectrometer are presented in Fig. 4 (A to C), with each experiment repeated three times and plotted with error bars. The estimated THG conversion efficiency is on the order of 10^−7^, depending on the incident pump power. Notably, at fundamental wavelength (FW) = 1685 nm (Fig. 4A), the THG response bends above the reference slope of 3 on a logarithmic scale, indicating dynamic enhancement as the resonance shifts into the pump wavelength. At FW = 1716 nm (Fig. 4B), the response bends below this slope, consistent with reduced resonant enhancement as the resonance shifts away from the pump. In contrast, the FW = 1756 nm (Fig. 4C) case remains close to cubic scaling, serving as a static-resonance reference. These opposite bending curvatures demonstrate that PIOT-induced resonance detuning can either enhance or suppress the apparent nonlinear power dependence, depending on the pump wavelength.

These changes in THG power dependence can be interpreted as PIOT modulation of the metasurface resonances. Specifically, the LC reorientation, which depends on the average laser power, modifies the effective nonlinear susceptibility $\chi^{\left(3\right)}$ of the metasurface. To accurately capture the intensity correlation, we extend the phenomenologically established nonlinear power law to include the dependence of the nonlinear susceptibility on the average power, $\chi^{\left(3\right)}=\chi^{\left(3\right)}\left(P_{\omega }^{a\mathrm{ve}}\right)\approx \chi_{0}^{\left(3\right)}+\chi_{1}^{\left(3\right)}P_{\omega }+\chi_{2}^{\left(3\right)}P_{\omega }^{2}+\cdots$. Here, $\chi_{1}^{\left(3\right)}$ and $\chi_{2}^{\left(3\right)}$ represent the effective third-order nonlinear susceptibility dependent on the incident average power, derived from a Taylor expansion of the original $\chi^{\left(3\right)}$ (detailed expansion and susceptibility definitions are in section S5). The corresponding expression for the TH power is then given below

$$P_{3\omega }=\chi_{0}^{\left(3\right)2}P_{\omega }^{3}+2\chi_{0}^{\left(3\right)}\chi_{1}^{\left(3\right)}P_{\omega }^{4}+\left(\chi_{1}^{\left(3\right)2}+2\chi_{0}^{\left(3\right)}\chi_{2}^{\left(3\right)}\right)P_{\omega }^{5}+2\chi_{1}^{\left(3\right)}\chi_{2}^{\left(3\right)}P_{\omega }^{6}+\chi_{2}^{\left(3\right)2}P_{\omega }^{7}$$ (3)

By assigning wavelength-dependent susceptibility values, we generate the purple fitting curves shown in Fig. 4 (A to C).

To quantify the power deviation from the static cubic response, we extract the output power difference, $\Delta P_{\text{out}}={\text{log}}_{10}P_{3\omega }^{\text{PIOT}}-{\text{log}}_{10}P_{3\omega }^{\text{static}}$, from both the experimental data in Fig. 4D, and the simulations in Fig. 4E. The experimental and simulated deviations show the same overall trend, although the simulated deviation appears at a lower pump power of ∼40 mW compared with ∼60 mW in the experiment. For FW = 1685 nm, $\Delta P_{\text{out}}$ is positive because the resonance shifts toward the pump wavelength and is attributed to the THG enhancement. For FW = 1716 nm, $\Delta P_{\text{out}}$ is negative because the resonance shifts away from the pump and reduces the resonant enhancement. For FW = 1756 nm, the deviation remains minimal, around −0.02, consistent with its near-cubic response. The oscillatory deviation is observed experimentally at powers up to 120 mW is not captured by the simulation and is likely associated with additional high-power effects not included in the model.

To further connect the THG modulation with the evolving fundamental resonance, we analyze the power-dependent multipolar contributions at the FW, as shown in Fig. 4F. In the figure, the circle size represents the normalized scattering cross section of each multipolar contribution, allowing the power-dependent evolution of the fundamental resonances to be compared on a common scale. Notably, the magnetic dipole contribution decreases with increasing incident power, whereas the electric dipole contribution exhibits the opposite trend. This behavior provides a qualitative explanation for the variation of efficiency enhancement in THG: At low power, the magnetic dipole resonance provides stronger field enhancement, while LC reorientation gradually weakens the magnetic dipole contribution and strengthens the electric dipole response.

### Noncubic dependence mechanism analysis

In this section, we further analyze the observed noncubic THG dependence by developing a nonlinear temporal coupled mode theory (*61*, *62*). This model links the PIOT-driven resonance detuning to the resonant field enhancement and the resulting nonlinear power-law modulation. It also provides insight into the time domain evolution of the fundamental and TH modes during the THG process.

To implement this model in our system, we consider three interacting modes: a magnetic dipole mode and an electric dipole mode at the fundamental frequency, and a nonlinear higher-order mode at the third-harmonic wavelengths of 561.67 and 572 nm, corresponding to the pump wavelengths of 1685 and 1716 nm, respectively. Because the magnetic and electric dipole modes have nearly orthogonal symmetries and distinct field distributions, their mutual coupling at the fundamental frequency is weak. We therefore reduce the system to an effective two-mode model consisting of one fundamental mode and one third-harmonic mode. Self- and cross-modulation terms are neglected to isolate the role of LC induced resonance detuning in the THG power-law response. Accordingly, this simplified model does not explicitly include LC-rotation saturation at high pump powers or additional local field enhancement effects that may arise after saturation

$$\begin{array}{c} \frac{\mathrm{da}}{\mathrm{dt}}=-\left(i\omega_{1}+\gamma_{1}\right)a+\sqrt{2\gamma_{1}}\;S^{1+}-i\text{κ}{\left(a^{*}\right)}^{2}b,\\ \frac{\mathrm{db}}{\mathrm{dt}}=-\left(i\omega_{2}+\gamma_{2}\right)b-i{\text{κ}}^{*}a^{3} \end{array}$$ (4)

where a and b denote the amplitudes of the fundamental and third-harmonic modes, respectively. Here, $\gamma_{1}$ and $\gamma_{2}$ are the decay rates of the modes, and κ is the effective third-order nonlinear coupling coefficient between the fundamental and third-harmonic modes. In the weak coupling regime, the system is driven by a narrowband Gaussian pulse defined as $S^{1+}\left(t\right)=G\left(t\right)\text{exp}\left[-i\omega_{i}t\right]$, where $G\left(t\right)=E_{i}\text{exp}\left[-\frac{t^{2}}{2\Delta t^{2}}\right]$. Here, G(t) is the slow varying envelope of the incidence, $E_{i}$ sets the peak envelope amplitude, and $\omega_{i}$ is the incident frequency. In our model, the central frequency $\omega_{1}$ and the damping rate $\gamma_{1}$ of the fundamental mode depend on the rotation angle of the LC, θ, while $\omega_{2}$ and $\gamma_{2}$ for the third-harmonic mode are treated as constants for simplicity. The numerical values of $\omega_{1},\omega_{2},\gamma_{1},\gamma_{2}$ and κ used in the calculations are provided in section S7.

**Fig. 5.**
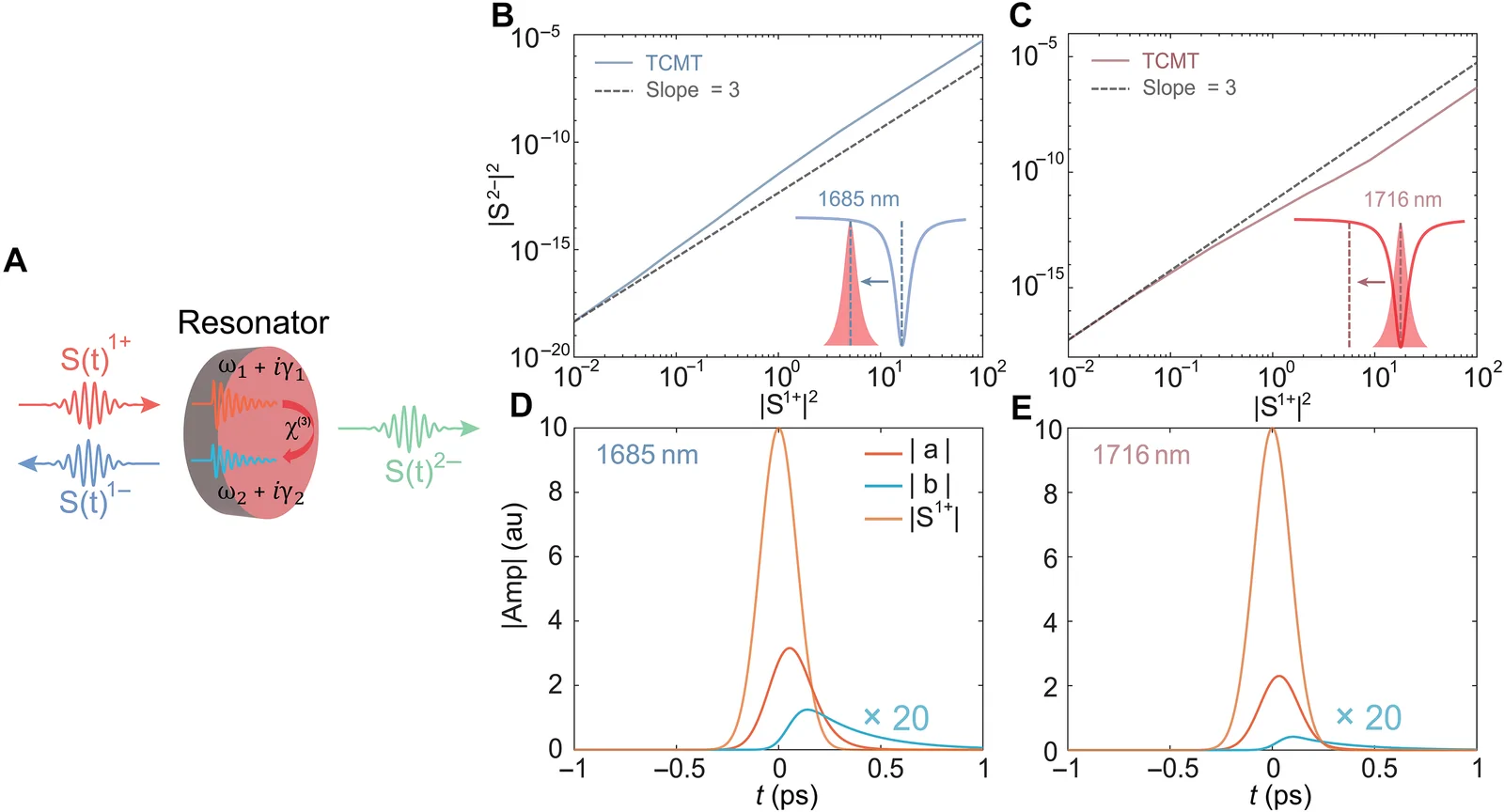
Theoretical demonstration of the bending curvature behavior in THG by temporal coupled mode theory. (**A**) Schematic of resonator nonlinear response. (**B**) illustrates the positive curvature bending due to the magnetic dipole resonance moving into the FW, and (**C**) shows negative curvature bending because the resonance is moving out of the FW. TCMT, temporal coupled mode theory. (**D** and **E**) Amplitudes of mode a and b at incident pulse amplitude $|S^{1+}\left(t\right)|=10$, where mode b is magnified 20 times for observation.

By extracting the absolute value of the output amplitude $|S^{2-}|$ from the calculation, we obtain the input and the output power relationship shown in Fig. 5. Figure 5A illustrates the schematic of the nonlinear response model, where the fundamental and TH modes are coupled through the third-order susceptibility $\chi^{\left(3\right)}$, allowing bidirectional (forward and backward) propagation. In this analysis, we consider only the forward coupling channel, corresponding to experimentally collected TH signal. Figure 5B illustrates the resonance evolution as the fundamental resonance shifts toward the pump wavelength. As the resonance approaches the pump wavelength, the TH output exhibits a rising trend with increasing input power, producing the up-bending curvature observed experimentally in Fig. 4A. Conversely, when the resonance initially overlaps with the pump and then shifts away, as shown in Fig. 5C, the resonant enhancement is progressively reduced, leading to the down-bending curvature observed in Fig. 4B.

Moreover, we further analyze the time-domain evolution of the FW amplitude a and the TH mode amplitude b under the input amplitude $|S^{1+}\left(t\right)|=10$, as shown in Fig. 5 (D and E). In the weak nonlinear coupling regime, the TH amplitude exhibits a much weaker yet clearly resolved temporal response than the FW amplitude and is magnified by a factor of 20 for visualization. This behavior confirms that the TH field is generated parametrically from the instantaneous FW amplitude through nonlinear coupling, rather than being independently excited. Although the FW temporal profiles in Fig. 5 (D and E) are similar, a pronounced difference is observed in the TH amplitude. This contrast highlights that the efficiency of harmonic generation is governed not only by the FW field strength but also by the spectral matching between the pump and the dynamically shifted fundamental resonance.

### Diffraction pattern modulation

**Fig. 6.**
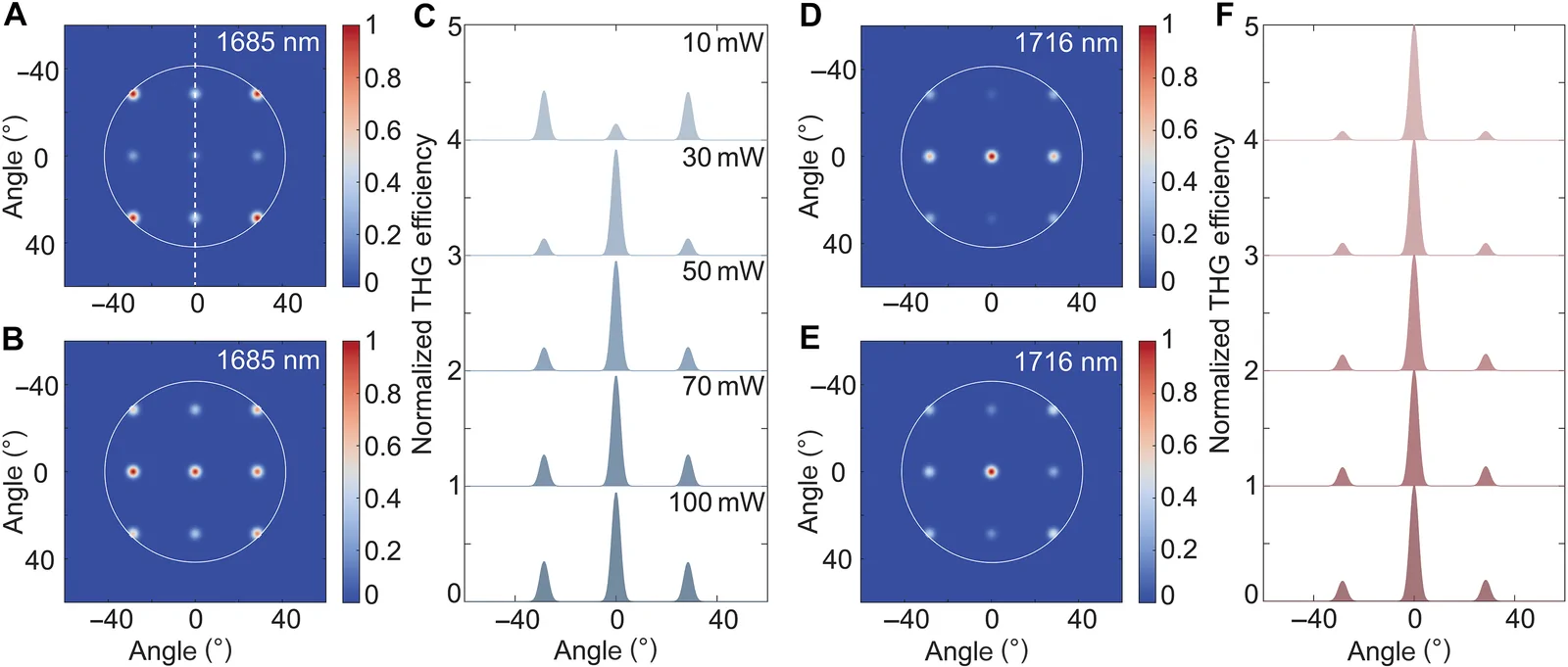
Power-dependent modulated TH back focal plane intensity patterns. (**A** and **B**) Simulated TH diffraction patterns at FW of 1685 nm with 10- and 100-mW pump power, respectively. (**C**) One-dimensional intensity plot of a vertical dash line at (A) with different powers. A clearly pattern modulation from 10 to 30 mW due to a high-order mode shift away from 3ω (ω=c/λ, where λ=1685 nm). (**D** and **E**) TH diffraction patterns for 10 mW and 100 mW pump power at FW of 1716 nm. (**F**) One-dimensional intensity plot from (D) to (E). Without being influenced by high-order modes, the TH intensity of 1716 nm demonstrates a limited modulation at order m,n=±1.

In this section, we numerically demonstrate a direct application of PIOT-driven THG tuning to nonlinear diffraction control. Beyond modulating the resonance line shape and conversion efficiency, this approach enables reconfigurable control of the nonlinear diffraction pattern. In the nonlinear regime, the diffraction pattern is predominantly governed by the spatial profile of the nonlinear mode, which can be evaluated from the far-field response derived from the TH near-field distribution. As the LC rotates between different states, the refractive-index change perturbs the optical mode at the TH wavelength, causing a resonance spectral shift. To obtain the power-dependent nonlinear diffraction patterns shown in Fig. 6, we explicitly include this LC-dependent TH mode evolution in the numerical model.

For each pump power, the PIOT-calculated LC director profile is converted into a spatially varying anisotropic permittivity tensor and used to calculate the resonant fundamental field at the selected pump wavelength. The resulting third-order nonlinear polarization at $3\omega_{1}$ is then used as the source for the TH simulation, yielding discrete Floquet diffraction orders m,n that are convoluted in the back focal plane with the angular spectrum of the finite Gaussian excitation beam.

Following this simulation procedure, we present results for the FF = 1685 nm and FF = 1716 nm, investigated in the previous section. Figure 6 (A and B) shows the diffraction patterns for input power 10 and 100 mW at FF = 1685 nm. A distinct redistribution is observed among the diffraction orders (m,n=±1), indicating the dynamic tuning effect. To guide the interpretation of the diffraction orders, the white circle in Fig. 6 (A, B, D, and F) marks the critical angle for total internal reflection. Diffraction orders outside this circle are confined within the substrate and do not radiate into the far field.

To quantify the modulation of each diffraction order, we extract a vertical cross section along the dashed white line in the two-dimensional pattern and plot the normalized intensity distribution in Fig. 6C. A pronounced enhancement, by nearly a factor of 6, of the zero-order diffraction is observed as the pump power increases from 10 to 30 mW. This behavior is attributed to mode shifting at the TH wavelength, as confirmed by the corresponding TH field profiles shown in section S8. The variation in the field distribution arises from LC reorientation, which effectively alters the mode profile and consequently the far-field diffraction pattern (detailed enhancement and suppression of each diffraction order are quantified in section S10). At this representative TH wavelength 1685/3=561.67 nm, the zero-order diffraction can be selectively suppressed or enhanced by tuning the incident pulse power. The TH pattern stabilizes above 30 mW, and further increases in power no longer affect the normalized intensity of the zero-order. However, instead, gradually enhance the first-order diffraction (m,n=±1). This contrast suggests that the modulation of the diffraction pattern in the nonlinear regime arises primarily from nonlinearly generated resonance tuning at the TH wavelength.

Similarly, Fig. 6 (D to F) displays the diffraction pattern variation at FF =1716 nm. In this case, the rotation of the LC causes only a slight variation in the ±1 diffraction intensities without altering the overall diffraction pattern, as shown in Fig. 6F. This weak modulation indicates that the nonlinearly generated multipolar content remains largely unchanged over the investigated pump-power range (see section S9 for details). In contrast to Fig. 6C, the modulation observed at 1685 nm arises mainly from the excitation of a resonant mode at the TH wavelength. Therefore, by engineering the mode state at the TH wavelength and varying the pump power, the optical field can simultaneously drive the LC to reorient and induce mode conversion at the TH wavelength, thereby achieving dynamic control over the far-field diffraction response.

## DISCUSSION

Our work establishes and validates an all-optical route to manipulate both the linear and nonlinear responses of LC-infiltrated silicon metasurfaces. Starting from a realistic metasurface platform, simulations and multipolar analysis identify two overlapping resonances, mode 1 (magnetic dipole) and mode 2 (electric dipole), whose fields sample the LC environment differently. A continuum model based on the optical Freedericksz transition describes the pump-induced rotation of the LC molecules, and the resulting orientation profiles are converted into an effective anisotropic refractive index using Euler rotation. This framework enables the prediction of PIOT-driven resonance dynamics. From a broader research perspective, spatially structured optical fields, particularly those with engineered polarization distributions, can generate spatially varying optical torque densities. This enables programmable LC reconfiguration beyond uniform intensity-driven actuation.

Linear measurements confirm a pronounced blue shift of the magnetic dipole mode by 36 nm and a minor red shift of the electric dipole mode by 3 nm. The polarization-dependent response rules out a thermal origin and indicates an experimental LC-rotation threshold on the order of tens of milliwatts, corresponding to an average intensity of 0.94 kW/cm^2^. In the nonlinear regime, power-dependent THG at three FWs exhibits characteristic “bending” in log-log plots: The THG response rises above the cubic power law when the resonance shifts toward the pump wavelength, whereas it is suppressed below the cubic trend when the resonance shifts away. The nearly static case follows the cubic scaling power law as a reference. Such tunable power-dependent polynomial nonlinear transfer functions in THG provide a promising route to reconfigurable optical nonlinearities for neuromorphic computational photonics, where adaptable activation functions and nonlinear mappings are central resources. A phenomenological fitting model with power-dependent effective $\chi^{\left(3\right)}$, combined with nonlinear TCMT, reproduces the observed trends and attributes them to resonance detuning induced by LC reorientation. In addition, the model captures the temporal response of the fundamental and TH modes, providing insight into the dynamical interplay between nonlinear excitation and resonant mode coupling.

Furthermore, far-field simulations reveal power redistribution within the TH diffraction pattern with varying LC orientations, including a nearly sixfold enhancement of the zero-order contribution and approximately twofold changes in selected higher-order channels, enabling selective enhancement or suppression of specific diffraction orders. These results verify the feasibility of optical torque–driven control of nonlinear emission in LC-assisted metasurfaces and link the incident energy, LC reorientation, resonance detuning, and nonlinear output. This framework provides quantitative design guidelines and practical relevance for tunable frequency conversion, nonlinear imaging, and multifunctional meta-devices, paving the way for applications of optically driven reconfigurable nonlinear photonic systems.

## MATERIALS AND METHODS

### Metasurface design

We designed the metasurface using the finite-element method implemented in COMSOL Multiphysics. In the simulations, the metasurface was excited by a normally incident plane wave from the backside of the substrate, and periodic boundary conditions were applied in the in-plane directions to model an infinite periodic array. The structure consists of a periodic array of crystalline silicon nanodisks on a quartz substrate, surrounded by an E7 LC layer and bounded by Teflon alignment layers that impose homeotropic LC alignment. The spatially varying LC rotation angle was calculated using the optical Freedericksz-transition model described in section S1 and then incorporated into the electromagnetic simulations through an anisotropic permittivity tensor. The unit-cell parameters, including the period, nanodisk radius, silicon thickness, Teflon thickness, and LC thickness, were set to P=955 nm, $h_{t}=150$ nm, $h_{\text{Si}}=245$ nm, $h_{\text{LC}}=2.85\;\mu$m, and R=350 nm, respectively.

### LC cell fabrication

To achieve homeotropic LC alignment, poly[4,5-difluoro-2,2-bis(trifluorome-thyl)-1,3-dioxole-*co*-tetrafluoroethylene], referred to as Teflon AF, was dissolved in octadecafluorodecahydronaphthalene at a concentration of 0.2% (*60*). The solution was applied by dip coating to both the cover glass and the metasurface substrate, dried at room temperature for 5 min, and subsequently baked at (60°C) for 10 min to remove residual solvent. The two substrates were then assembled into an LC cell, and E7 LC was infiltrated into the gap by capillary action. This treatment provides homeotropic boundary conditions on both sides of the LC layer, orienting the LC director perpendicular to the metasurface plane in the initial state.

### Experimental setup for metasurface characterizations

The experimental setups for both linear optical characterization and nonlinear THG measurements are described in section S3. For the linear measurements, a broadband white light source was used to probe the transmission spectra of the LC-infiltrated metasurface while a spectrally detuned femtosecond pump beam drove the polarization-induced LC reorientation. For the nonlinear measurements, the white light probe was removed, and the same femtosecond laser was tuned to the fundamental resonance wavelengths to simultaneously drive LC reorientation and excite THG from the silicon metasurface.
