# Supplementary material for: All-optical control of nonlinear emission from resonant metasurfaces
Source: Sci Adv. 2026 Sep 25;12(39):eaeh0904. doi: 10.1126/sciadv.aeh0904 (PMC13614378; doi:10.1126/sciadv.aeh0904)
Supplement: Supplementary file 1 [file sciadv.aeh0904_sm.pdf]

Science Advances
AAAS

Supplementary Materials for

All-optical control of nonlinear emission from resonant metasurfaces

Ziwei Yang *et al.*

Corresponding author: Mohsen Rahmani, mohsen.rahmani@ntu.ac.uk;
Dragomir Neshev, dragomir.neshev@anu.edu.au

Sci. Adv. **12**, eaeh0904 (2026)
DOI: 10.1126/sciadv.aeh0904

This PDF file includes:

Supplementary Text
Figs. S1 to S10
Tables S1 and S2

Supplementary Text

SI. 1: Theory of Polarization induced optical torque LC distribution

In order to demonstrate the spatial distribution of the LC orientation by PIOT, we provide the full equation derivation, which can illustrate the evolution of the optical Freedericksz transition under optical force (*57*).

We first started with a standard Euler-Lagrange equation, which is commonly used to describe the reorientation of the director axis under the LC system,

$$\frac{\partial F}{\partial \theta} - \frac{d}{dz}\frac{\partial F}{\partial \theta'} = 0, \tag{S1}$$

where $\mathbf{F} = \mathbf{F}_d + \mathbf{F}_{\text{op}}$ denotes the free energy of the whole system. And $\mathbf{F}_d$ is the distortion energy, and $\mathbf{F}_{\text{op}}$ stands for the optical dipolar interaction energy. By describing the distortion energy with three different elastic constants K_1, K_2 and K_3, we can extend the distortion term $\mathbf{F_d}$ and the optical dipolar interaction term $\mathbf{F}_{\text{op}}$ as following;

$$F_d = \frac{1}{2}K_1(\nabla\cdot\hat{\mathbf{n}})^2 + \frac{1}{2}K_2(\hat{\mathbf{n}}\cdot\nabla\times\hat{\mathbf{n}})^2 + \frac{1}{2}K_3(\hat{\mathbf{n}}\times\nabla\times\hat{\mathbf{n}})^2 \tag{S2}$$

$$F_{\text{op}} = -\frac{1}{4\pi}\int \mathbf{D}\cdot d\mathbf{E} = -\frac{\varepsilon_\perp}{8\pi}E^2 - \frac{\varepsilon_a}{8\pi}\left\langle(\hat{n}\cdot\mathbf{E})^2\right\rangle, \tag{S3}$$

The $\mathbf{E}$ is the optical field, and $\langle\rangle$ gives the time average for the optical electric field at ultrahigh frequency. And $\varepsilon_a = \varepsilon_\parallel - \varepsilon_\perp$, $\varepsilon_\parallel$ is the permittivity parallel to the LC director $\hat{\mathbf{n}}$, and $\varepsilon_\perp$ is perpendicular to the director.

For simplicity, we consider the driving field as a sinusoidal plane wave, and the Freedericksz transition happens only in the $x-z$ plane. Then, we obtain the electric field $\mathbf{E} = E_x\hat{x} + E_z\hat{z}$, and only K_1, K_3 is taken into account in the transition. In addition, defining $\hat{n} = \sin\theta\hat{x} + \cos\theta\hat{z}$, we rewrite the two force terms S2 and S3 as follows:

$$F_d = \frac{1}{2}(K_1\sin^2\theta + K_3\cos^2\theta)\left(\frac{d\theta}{dz}\right)^2 \tag{S4}$$

$$F_{\text{op}} = -\frac{\varepsilon_\perp}{16\pi}(|E_x|^2 + |E_z|^2) - \frac{\Delta\varepsilon}{16\pi}\left[\sin^2\theta|E_x|^2 + \cos^2\theta|E_z|^2 + 2\sin\theta\cos\theta\,\Re(E_xE_z^*)\right] \tag{S5}$$

Then, we substitute S4 and S5 into S1 to achieve the general LC optical rotation Euler-Lagrange equation,

$$
\begin{aligned}
&\left(K_1 \sin^2\theta + K_3 \cos^2\theta\right) \frac{d^2\theta}{dz^2} - (K_3 - K_1)\sin\theta\cos\theta\left(\frac{d\theta}{dz}\right)^2 \\
&+\frac{\Delta\varepsilon}{16\pi}\left[\sin 2\theta\left(|E_x|^2 - |E_z|^2\right) + \cos 2\theta\,\Re(E_x E_z^*))\right] = 0.
\end{aligned}
\tag{S6}
$$

where $\theta = \theta(z)$.

In our configuration, the LC is homeotropically aligned with the metasurface, and the driving electric field of the laser is linearly polarized along the $x-$ axis ($E_z = 0$). Under these conditions, Eq. S6 can be further simplified as shown in Eq. S7,

$$
K\frac{d^2\theta}{dz^2} + \frac{\Delta\varepsilon}{16\pi}\sin 2\theta|E_x|^2 = 0. \tag{S7}
$$

Converting this expression into SI units yields:

$$
K\frac{d^2\theta}{dz^2} + \frac{\varepsilon_0\Delta\varepsilon}{4}\sin 2\theta|E_x|^2 = 0. \tag{S8}
$$

To estimate the optical intensity required to trigger a noticeable LC reorientation, we apply a simplified small-angle torque-balance analysis to Eq. (S8). For $\theta \ll 1$, we use $\sin(2\theta) \approx 2\theta$, which yields a linearized equation,

$$
K\frac{d^2\theta}{dz^2} + \frac{\varepsilon_0\Delta\varepsilon}{2}2\theta|E_x|^2 = 0. \tag{S9}
$$

A simple order-of-magnitude estimate can be obtained by balancing the elastic and optical terms across the thickness of LC cell h_{LC}. The curvature of the director profile can then be approximated as $d^2\theta/dz^2 \sim \theta/h_{LC}^2$. Then the corresponding characteristic onset field is given by,

$$
|E_{th}|^2 \approx \frac{2K}{\varepsilon_0\Delta\varepsilon\, h_{LC}^2}. \tag{S10}
$$

The corresponding incident intensity is obtained from $I = \frac{1}{2}n\varepsilon_0 c|E_x|^2$, where n is the LC refractive index at the pump wavelength. Using representative parameters from the LC datasheet, $K = 11.1\ pN$, $n = 1.5$, $d = 27.7\ \mu m$ and $\Delta\varepsilon = 0.72$, the estimated threshold intensity is approximately $0.896\ \mathrm{k}W/cm^2$.

Experimentally, the reorientation threshold is observed at a pump power of about $P_{\mathrm{th}} \approx 60$ mW. With an effective illuminated area of roughly $80\ \mu m \times 80\ \mu m$, the corresponding average incident intensity is about $0.9375\ \mathrm{kW}/cm^2$. This value is slightly higher than the theoretical estimate, which is reasonable given the simplified nature of the model and possible experimental factors such as beam nonuniformity and material parameter uncertainties.

After solving the LC rotation angle along the light propagation direction, we implement the spatial distribution of LC in the metasurface unit cell model. By using the Euler coordinate transformation shown below, we can import the spatially variant LC permittivity into the simulation system.

$$M_{LC} = \varphi \cdot \theta \cdot \varepsilon \cdot \theta^{-1} \cdot \varphi^{-1} \tag{S11}$$

where φ is the in-plane rotation and θ comes from the optical Freedericksz transition in the $x - z$ plane.

The general expansion is shown as follows.

$$M_{LC} = \begin{pmatrix} \varepsilon_{yy}\sin(\phi)^2 + \cos(\phi)\,\sigma_2 & \sin(\phi)\,\sigma_2 - \sigma_4 & \varepsilon_{zz}\cos(\phi)\cos(\theta)\sin(\theta) - \varepsilon_{xx}\cos(\phi)\cos(\theta)\sin(\theta) \\ \cos(\phi)\,\sigma_1 - \sigma_4 & \varepsilon_{yy}\cos(\phi)^2 + \sin(\phi)\,\sigma_1 & \varepsilon_{zz}\cos(\theta)\sin(\phi)\sin(\theta) - \varepsilon_{xx}\cos(\theta)\sin(\phi)\sin(\theta) \\ -\cos(\phi)\,\sigma_3 & -\sin(\phi)\,\sigma_3 & \varepsilon_{xx}\cos(\theta)^2 + \varepsilon_{zz}\sin(\theta)^2 \end{pmatrix} \tag{S12}$$

where σ_1, σ_2, σ_3, and σ_4 are defined as

$$\begin{aligned} \sigma_1 &= \varepsilon_{zz}\sin(\phi)\cos(\theta)^2 + \varepsilon_{xx}\sin(\phi)\sin(\theta)^2 \\ \sigma_2 &= \varepsilon_{zz}\cos(\phi)\cos(\theta)^2 + \varepsilon_{xx}\cos(\phi)\sin(\theta)^2 \\ \sigma_3 &= \varepsilon_{xx}\cos(\theta)\sin(\theta) - \varepsilon_{zz}\cos(\theta)\sin(\theta) \\ \sigma_4 &= \varepsilon_{yy}\cos(\phi)\sin(\phi) \end{aligned} \tag{S13}$$

SI. 2: LC fabrication methodology

To achieve homeotropic alignment of LC, the solution of Poly[4,5-difluoro-2,2bis(trifluoromethyl)-1,3-dioxole-co-tetrafluoroethylene], referred to as Teflon AF, is dissolved in octadecafluorodecahydronaphthalene (CAS 37626-13-4 and 30694-5) with 0.2% (*60*). The solution was applied by dip coating and was then dried for 5 minutes at room temperature, after which it was baked at 60^oC for another 10 minutes to remove residual solvent. This surface treatment induced vertical (homeotropic) alignment of the LCs. In particular, Teflon AF is an amorphous fluoropolymer and differs from crystalline polytetrafluoroethylene (PTFE), which is conventionally employed for planar alignment.

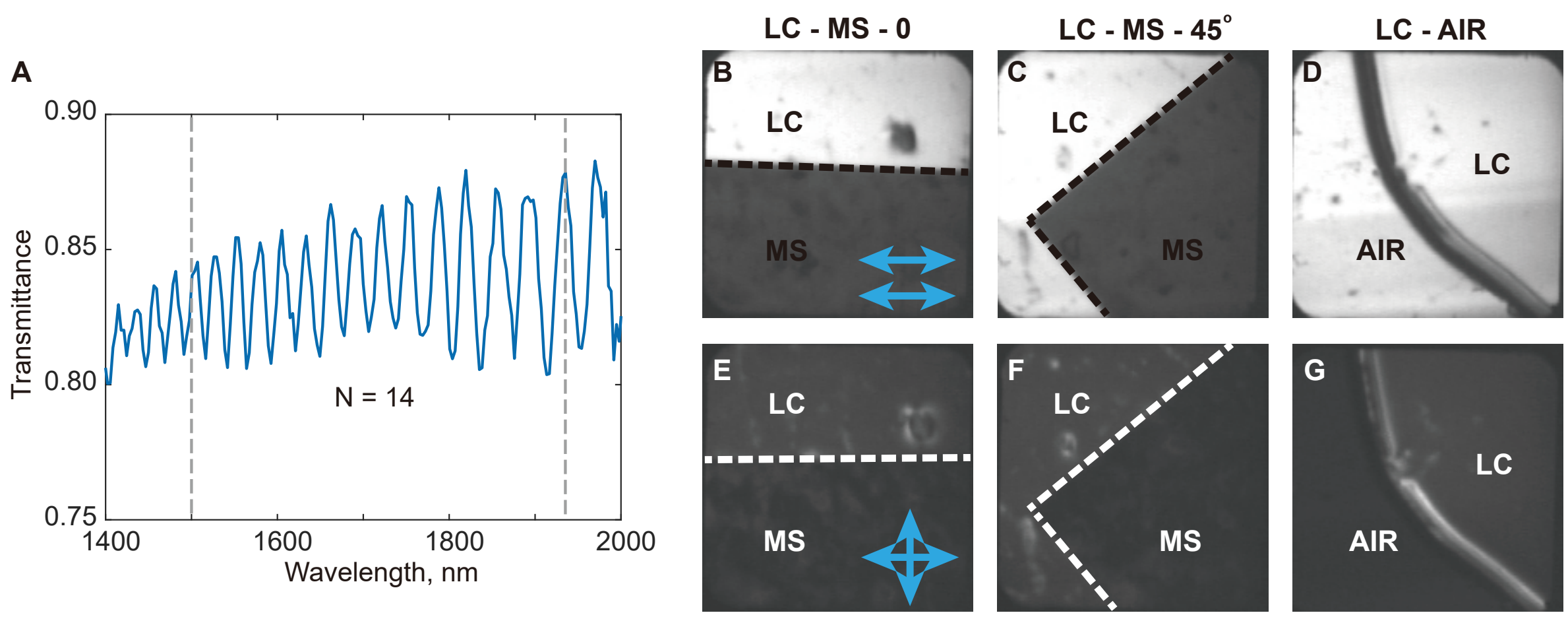

Figure S1: **LC cell fabrication characterization** (**A**) Transmittance of the empty LC cell, the fringes are from the Fabry-Perot interference. The fringes number $N = 14$, counting from $\lambda_1 = 1300\ nm$ to $\lambda_1 = 1935.43\ nm$. (**B-G**) are the microscope images of the LC metasurface (labelled as MS), co-polarization is applied for (**B-D**) and cross-polarization states are present in (**E-G**). (**B**) and (**E**) show the sample edge that is parallel to the one polarizer direction, and (**C**) and (**F**) illustrate the 45° angle to the polarizer. (**D**) and (**G**) are the interface between LC and Air.

Before infiltrating the LC, we measured the thickness of the LC cell, shown in Fig. S1(A). Counting the number from the Fabry-Perot interference in the transmittance setup, we determined the actual thickness of the LC cell to be $h_{LC} = 27.7\ \mu$m in reality. After infiltration, the fabricated LC-metasurface is observed under an optical microscope to check the homeotropic condition

of the LC molecules. Firstly, two polarizers are utilized to define the polarization state. Figures Fig. S1(B-D) show that the two polarizers are parallel to each other, while Fig. S1(E-G) depicts the cross-polarization situations. From the figure, we can observe that the light intensity remains stable during sample rotation under different polarized states, as shown in Fig. S1(B), (C), (E), and (F), indicating the homogeneity of the LC. Even in comparison to air, as illustrated in Fig. S1(D) and (G), the LC demonstrates transparency and is well aligned.

SI. 3: Characterization setup

Linear optical characterization: The experimental setup for linear optical characterization is illustrated in this section, Fig. S2. A femtosecond laser with a pulse duration of 150 fs and a central wavelength of 1450 nm serves as the driving source. The beam first passes through a linear polarizer to define its polarization state, followed by a pair of lenses that expand the beam size. A band-pass filter is then used to suppress unwanted noise signals originating from the laser. A beam splitter couples the probe light, which is generated by a white-light lamp. To minimize scattering from the LC inhomogeneities, illumination is directed from the backside of the metasurface. The transmitted signal is collected using a 10 times objective lens, which only collects light of normal incidence. Two additional lenses are used to confine the illumination area to the region of interest on the sample. Finally, the image is relayed through a tube lens to a camera for visualization, and the spectral signal is recorded by a fiber-coupled spectrometer (Ocean Optics).

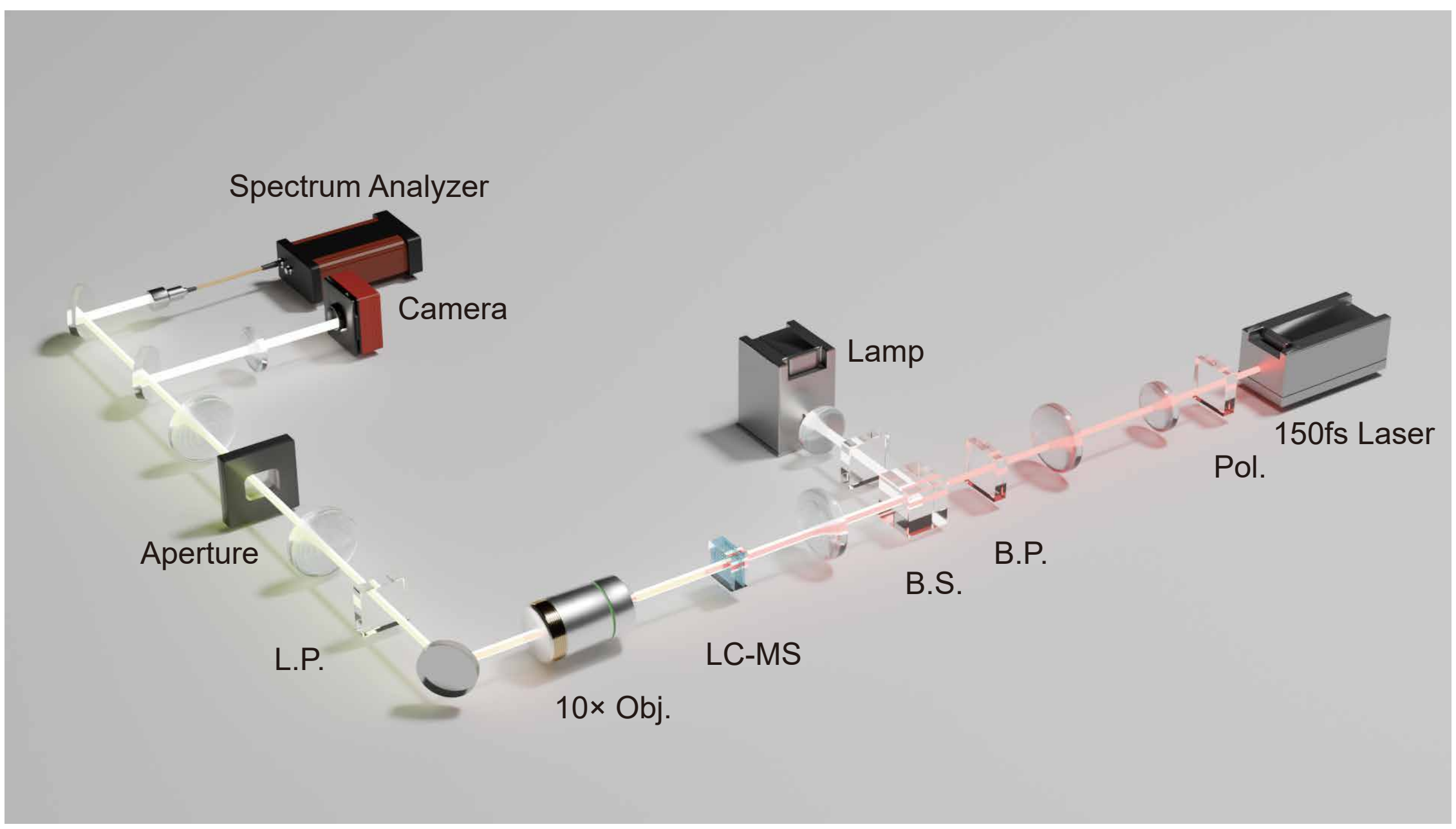

Figure S2: **Linear characterization setup**

Nonlinear optical characterization: For the nonlinear characterization, Fig. S3, we use a setup similar to that used for the linear characterization, except that the broadband white-light source, confining aperture, and two relay lenses are removed. The long-pass filter used in the linear characterization is replaced by a short-pass filter to remove the high-power incident laser and

protect the spectrometer during THG measurement. The incident femtosecond laser is tuned to the fundamental resonance wavelength. The generated TH signal is collected using a 10× objective, which mainly collects the zeroth-order nonlinear emission. As the incident pump power increases, LC rotation occurs accordingly, modifying the metasurface resonance condition and leading to the power-dependent THG response discussed in the main text. Fig. S2 and Fig. S3 are created by Blender 3.1.0 using an optical components pack provided by Ryo Mizuta Graphics.

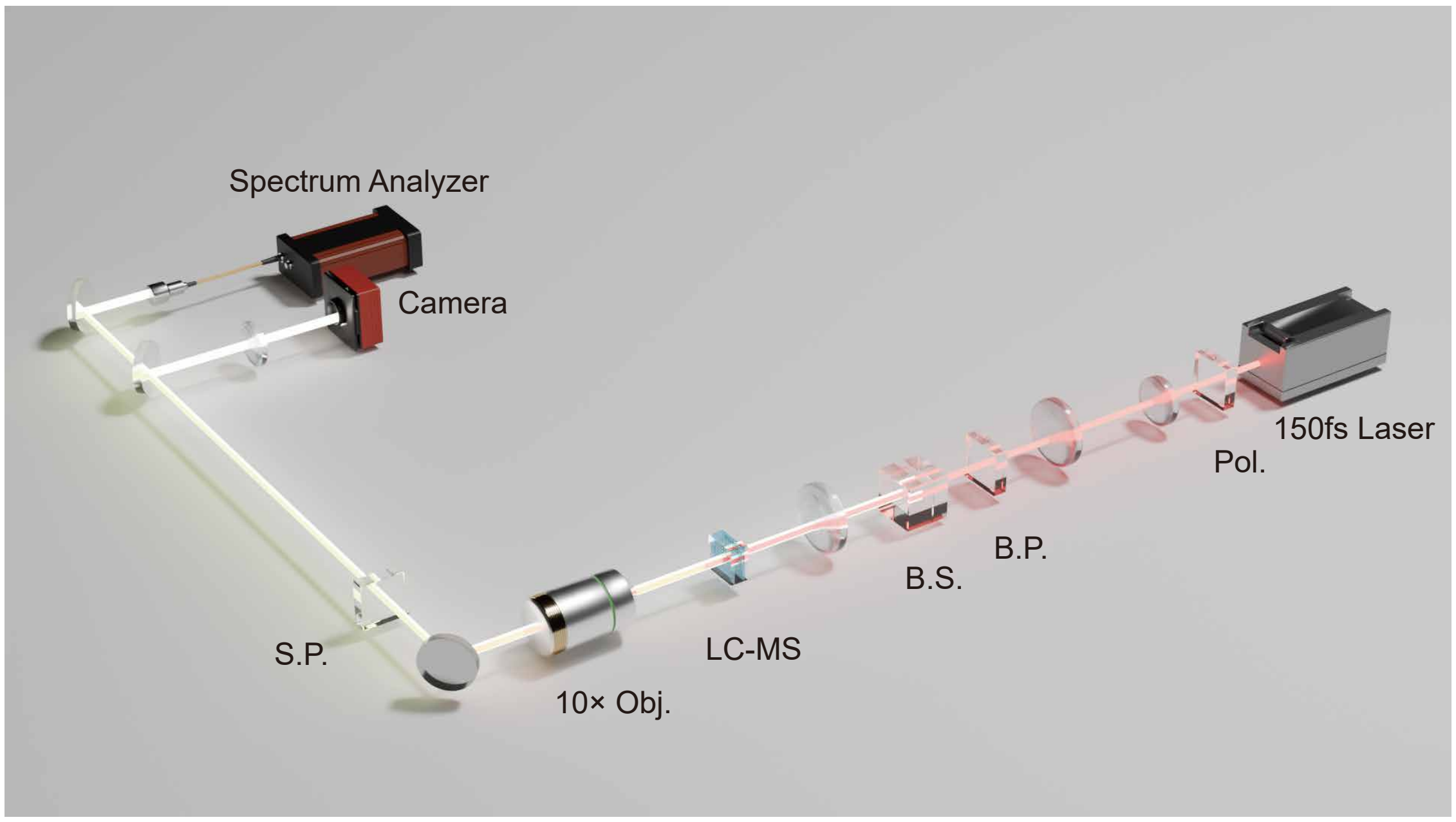

Figure S3: **Nonlinear characterization setup**

Choice of THG in silicon metasurfaces: THG was chosen as the nonlinear process in this silicon metasurface platform for three main reasons. First, crystalline silicon is centrosymmetric and therefore does not support bulk electric-dipole SHG. Any SHG signal would mainly originate from surface or interface symmetry breaking, making it difficult to distinguish surface-related nonlinear effects from the resonance tuning induced by PIOT-driven LC reorientation. In contrast, THG arises from the intrinsic third-order nonlinearity of silicon and therefore provides a more direct nonlinear channel for demonstrating PIOT-controlled nonlinear emission. Second, the experiment is performed in the near-infrared spectral range, where silicon has relatively low optical absorption. This helps reduce thermal buildup, which is important because heating can also modify the LC response and obscure the optical-torque-driven effect. Third, silicon metasurfaces provide a widely

used and experimentally accessible nonlinear photonic platform, and THG is a representative nonlinear response in such systems. Therefore, the use of THG allows us to demonstrate LC-based all-optical nonlinear tunability without relying on non-centrosymmetric materials or additional symmetry-broken nonlinear structures.

SI. 4: Control measurements for linear and nonlinear characterization

Linear characterization with two-polarization comparison: After setting up the linear experimental setup, we measure the linear transmittance with different input polarizations of the probe beam; refer to Fig. S4(A). With the horizontal polarization of the probe beam, labeled as H, we observe a clear resonance shift from both the MD dominant mode 1 and the ED dominant mode 2, with a clear opposite direction shift as the pump power increases. This phenomenon indicates the anisotropy of the LC orientation and not the thermal effect, which would shift the ED and MD modes in the same direction.

Further proof comes from the rotation of the probe polarizer to the V state, as shown in the second figure in Fig. S4(A). For the V state, mode 1 also experiences a refractive index variation with an insensitive blue shift of the resonance, but mode 2 remains constant because the orientation of the LC does not influence the V state. Therefore, the second figure in Fig. S4(A) illustrates a relatively stable state for mode 1 and mode 2. To compare the transmittance variation between the two modes, we demonstrate the ΔT, % in Fig. S4(B). From this figure, we observe the largest variation occurring approximately in the resonance shift region from 1716 nm to 1685 nm with a $\approx$ 10% difference.

This result reveals that the modulation of the LC is a result of the PIOT effect under the viscosity of the LC in contrast to the thermal effect as in Ref. (*20*).

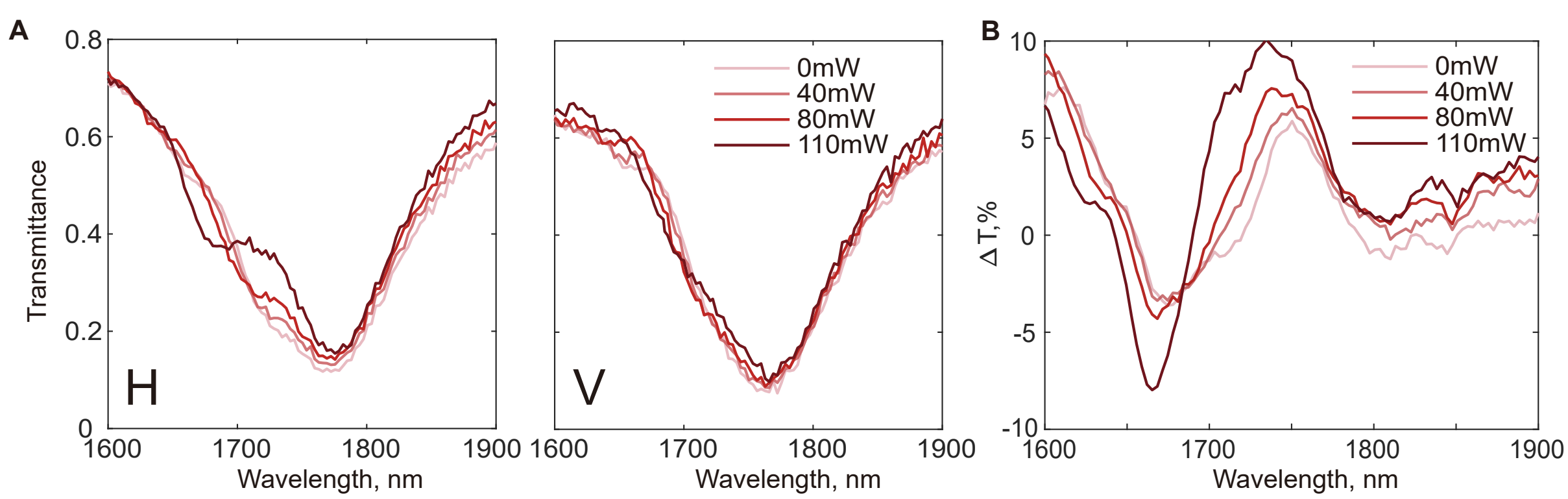

Figure S4: **Linear characterization for H and V polarization state and its differences**

Explanation of the discrepancy between measurement and simulation: As noted in the main text, the comparison between measurement and simulation shows that mode 1 is less pronounced

experimentally than mode 2. This discrepancy may arise from the stronger sensitivity of the magnetic-dipole-dominated mode to the surrounding LC environment. Since this mode relies on circulating displacement currents, it is more susceptible to additional damping introduced by the LC, the Teflon alignment layer, and fabrication-related interfacial imperfections. In contrast, the electric-dipole-dominated mode is more localized around the silicon resonator and is therefore less affected by LC-induced perturbations, particularly under surface-anchored LC alignment.

Using a simple analytical expression for field transmittance, we can represent this relationship via the equation:

$$t(\omega) = 1 + \frac{2i\gamma_{r,e}\omega}{\omega_e^2 - \omega^2 - 2i(\gamma_{r,e} + \gamma_{nr,e})\omega} + \frac{2i\gamma_{r,m}\omega}{\omega_m^2 - \omega^2 - 2i(\gamma_{r,m} + \gamma_{nr,m})\omega}. \tag{S14}$$

Here, e and m denote the electric- and magnetic-dipole-dominated modes, respectively, while γ_r and γ_{nr} represent the radiative and non-radiative damping rates. To describe the reduced visibility of the magnetic-dipole resonance in the experiment, we introduce an additional disorder-induced non-radiative damping term only for the magnetic mode:

$$\gamma_{nr,m}(D) = \gamma_{nr,m}^0 + \eta_{\text{dis}} D \gamma_{\text{total},m}^0. \tag{S15}$$

Therefore, the magnetic-mode contribution becomes,

$$t(\omega) = 1 + \frac{2i\gamma_{r,e}\omega}{\omega_e^2 - \omega^2 - 2i(\gamma_{r,e} + \gamma_{nr,e})\omega} + \frac{2i\gamma_{r,m}\omega}{\omega_m^2 - \omega^2 - 2i\gamma_{\text{total},m}(D)\omega}. \tag{S16}$$

The parameter D quantifies the effective disorder strength, while describes how efficiently this disorder contributes to magnetic-mode damping. Based on this phenomenological formulation, the Fig S5(A) below shows that the magnetic-dipole-dominated resonance peak is significantly less pronounced than the electric-dipole-dominated resonance peak. By introducing additional loss into the alignment layer or in the near-field liquid crystal region, the same trend can also be reproduced using numerical simulation, as shown in Fig S5(B). These results suggest that the weaker contrast observed in Mode 1 mainly arises from extra damping and interfacial defects, which are not accounted for in the idealized simulation.

Control measurement of the THG signal origin: To verify that the measured THG signal mainly originates from the resonant silicon metasurface, we performed a control measurement at an off-resonant pump wavelength. As shown by the purple curve in Fig. S6, when the pump wavelength

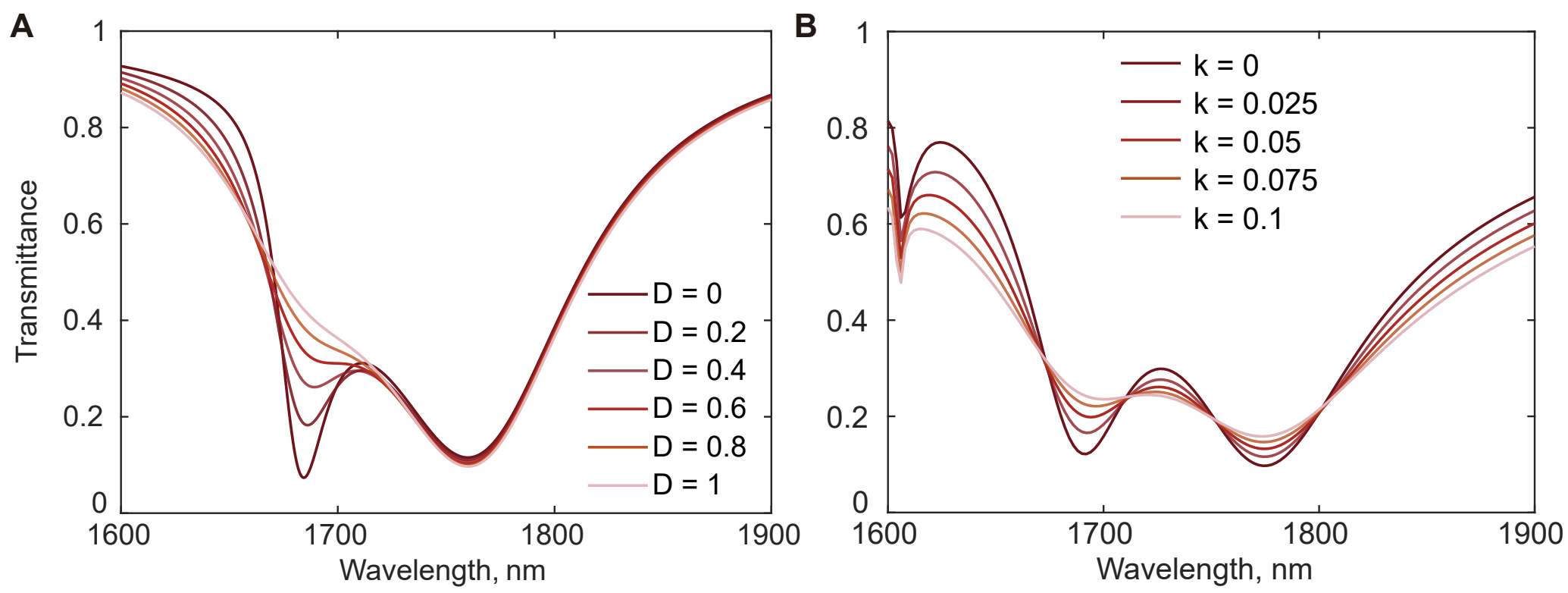

Figure S5: **Linear characterization for H and V polarization state and its differences**

is set to 1600 nm, far away from the metasurface resonance, only a weak THG signal becomes detectable at pump powers above approximately 220 mW. This off-resonant signal remains much weaker than the resonantly enhanced THG signal measured at 1715 nm. The comparison indicates that the background contribution from the LC, substrate, and other non-resonant components is significantly weaker than the THG generated by the resonant silicon metasurface.

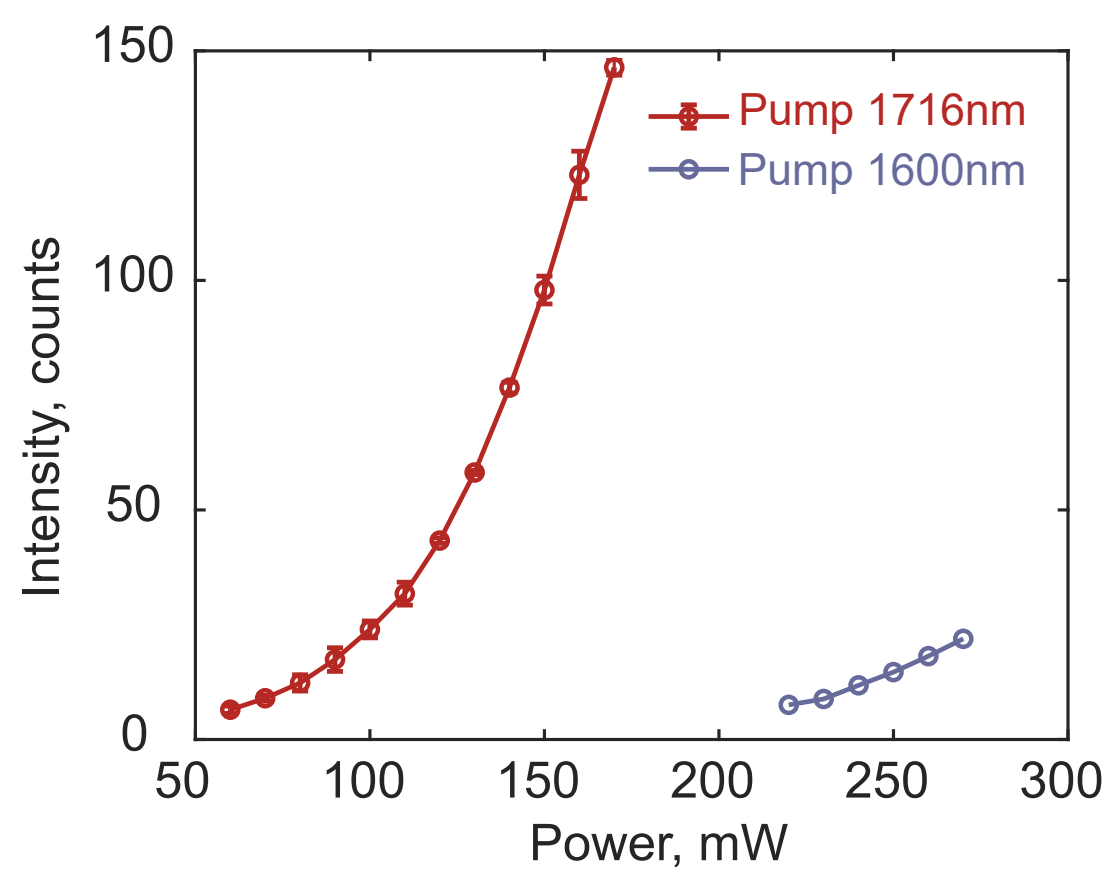

Figure S6: **Power-dependent THG signals under resonant and non-resonant pumping**

In this control experiment, the pump power was increased up to 270 mW. To avoid possible sample damage, higher pump powers were not used. Since the off-resonant THG signal remains substantially weaker than the resonant THG signal even at this high pump power, we conclude that the THG response observed in the main experiment is primarily generated by the resonantly enhanced silicon metasurface.

SI. 5: The derivation of the nonlinear fitting equation and its coefficient dispersion relation

Here, we start with the general equation for third-harmonic generation.

$$\mathbf{P}_{3\omega}^{(3)} = \eta(\chi^{(3)})^2\mathbf{P}_{\omega}^3 \tag{S17}$$

where η is the effective coefficient of THG conversion, which is proportional to the mode enhancement and also the reciprocity of the refractive index in the FW and TH regions. Conventionally, the third-order nonlinear susceptibility remains constant, denoted as $\chi_0^{(3)}$, with a cubic relation between the input and output power. However, in our case, the light induced LC rotation effect can influence the behavior of the TH conversion. Therefore, we introduce a power-dependent TH with Taylor expansion as follows;

$$\chi^{(3)} = \chi_0^{(3)} + \chi_1^{(3)}P_\omega + \chi_2^{(3)}P_\omega^2 + \cdots \tag{S18}$$

for the $\chi_1^{(3)}P_\omega$ term, it fits well into the low power deviation regime, which mainly describes the effect of dynamic resonance involvement. The third term $\chi_2^{(3)}P_\omega^2$ can describe the deviation that occurs in the high power regime. Then, we substitute S18 into S17 to get the expansion equation as;

$$P_{3\omega} = \eta\left[\chi_0^{(3)2}P_\omega^3 + 2\chi_0^{(3)}\chi_1^{(3)}P_\omega^4 + \left(\chi_1^{(3)2} + 2\chi_0^{(3)}\chi_2^{(3)}\right)P_\omega^5 + 2\chi_1^{(3)}\chi_2^{(3)}P_\omega^6 + \chi_2^{(3)2}P_\omega^7\right] \tag{S19}$$

As illustrated by the related efficient susceptibilities $\chi_0^{(3)}$, $\chi_1^{(3)}$, and $\chi_2^{(3)}$ in Fig. S7, we observe the dispersion relation of susceptibilities between 1680 nm and 1760 nm, calculated from the S19. It is clear that the $\chi_0^{(3)}$ term is still dominant, which is around 3 orders of magnitude larger than the $\chi_1^{(3)}$ and $\chi_2^{(3)}$. As expected, the positions of the resonances, ED and MD dominant modes 1 and 2, illustrate a broad enhancement effect compared with 1680 nm, which is off resonance.

Moreover, $\chi_1^{(3)}$ presents the curvature of the up or down bending, depending on the sign of the susceptibilities; the value also denotes the level of deviation. In the meantime, $\chi_2^{(3)}$ shows the curvature in the high power regime; however, it demonstrates a small value in the simulation due to the absence of high power effects added in. It shows a comparable value of $\chi_1^{(3)}$ in the experiment.

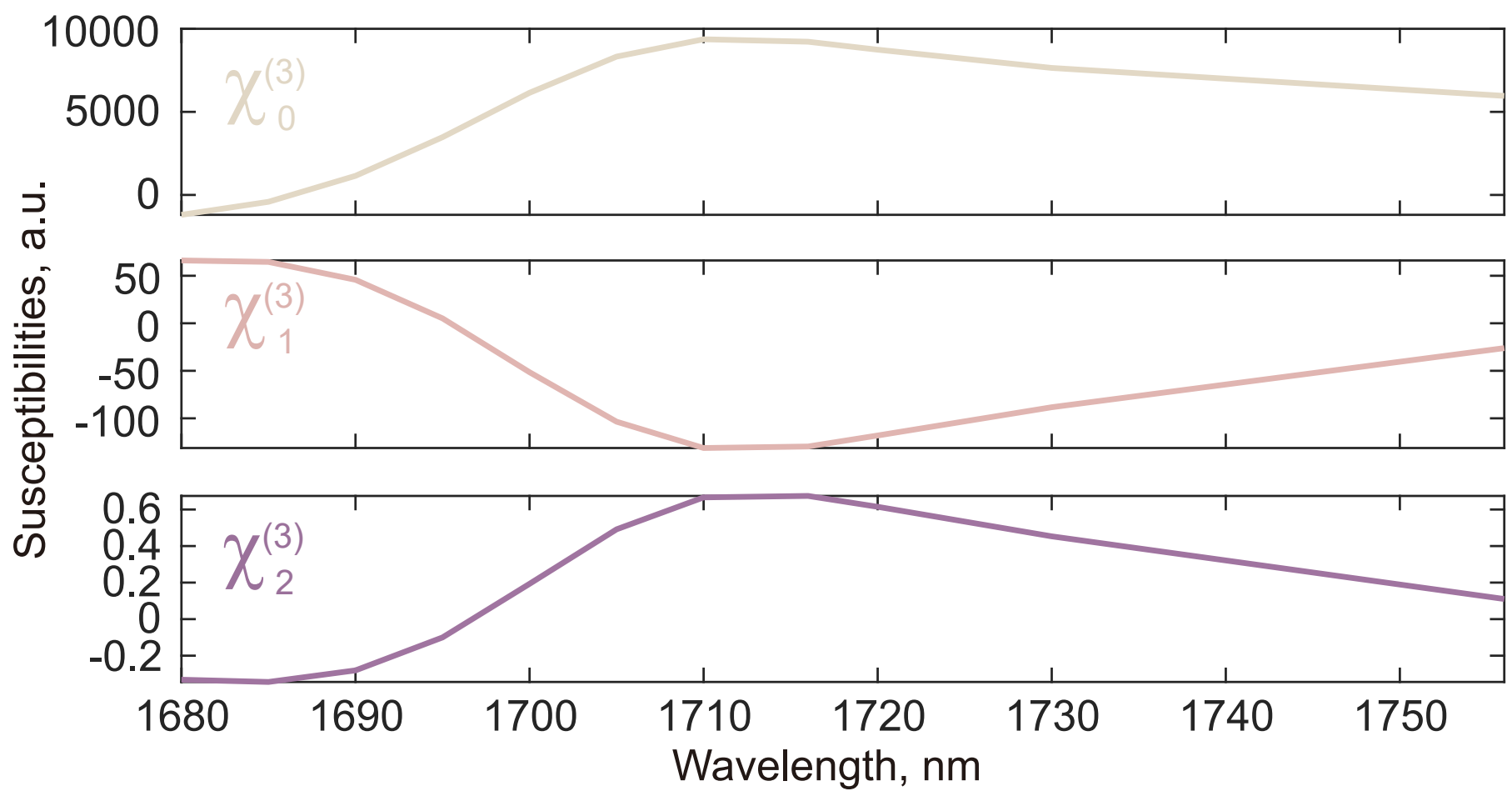

Figure S7: **Dispersion relation of fitting $\chi_0^{(3)}$, $\chi_1^{(3)}$ and $\chi_2^{(3)}$**

SI. 6: High-power THG response after PIOT-induced detuning saturation

To further clarify the high-power THG behavior, we extend the THG response for the three pump wavelengths in the high-power regime. As shown in Fig. S8, the enhanced and suppressed bending cases gradually approach the cubic reference when the incident pump power exceeds approximately 110 mW. This behavior indicates that the PIOT-induced resonance-detuning process becomes saturated at high pump power. Once the LC reorientation reaches a near-saturated state, the resonance position no longer changes with further increases in pump power. As a result, the metasurface behaves approximately as a static nonlinear resonator, and the THG signal recovers the conventional cubic power dependence.

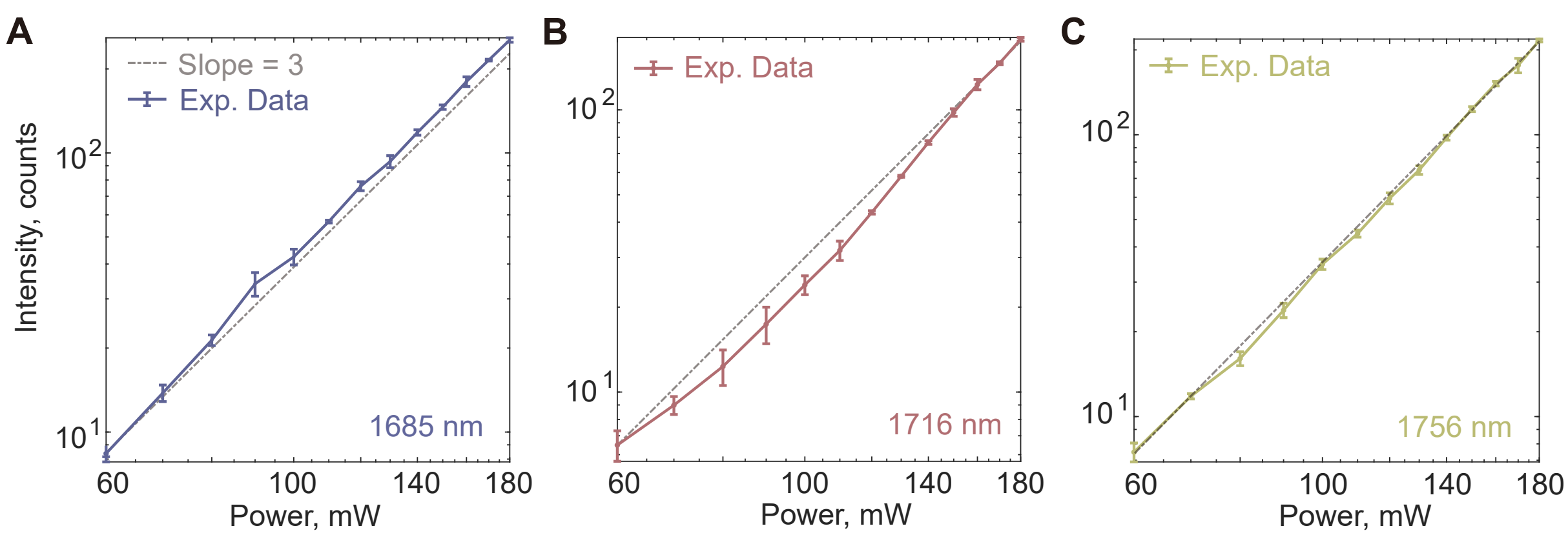

Figure S8: **Recovery of cubic THG scaling in the high-power regime.**

SI. 7: Implementation of nonlinear temporal coupled mode theory

In this section, we provide the implementation details of the effective nonlinear temporal coupled-mode theory (NTCMT) model used in the main text to describe the PIOT-induced non-cubic THG response. The model is formulated as a phenomenological two-mode description, consisting of one effective fundamental resonance and one effective third-harmonic resonance. Its purpose is not to predict the absolute THG conversion efficiency from first-principles nonlinear overlap integrals, but to capture how LC-induced resonance detuning modifies the input-output power dependence of the generated TH signal.

This form follows the standard structure of nonlinear temporal coupled-mode descriptions of resonant THG (*61, 62*), but the coupling coefficient is treated here as an effective fitting parameter. Beginning with the model shown in the main text,

$$\begin{aligned}\frac{da}{dt} &= -(i\omega_1 + \gamma_1)a + \sqrt{2\gamma_1}\, S^{1+} - i\kappa(a^*)^2 b,\\ \frac{db}{dt} &= -(i\omega_2 + \gamma_2)b - i\kappa^* a^3,\end{aligned} \tag{S20}$$

Here, a and b denote the modal amplitudes of the fundamental and third-harmonic resonances, respectively. The parameters ω_1 and ω_2 are the resonance angular frequencies of the fundamental and third-harmonic modes, while γ_1 and γ_2 represent their corresponding total decay rates. S^{1+} is the incident pump-wave amplitude coupled into the fundamental mode. The coefficient κ describes the effective third-order nonlinear coupling between the fundamental and third-harmonic modes. In this phenomenological model, κ is treated as a fitting parameter that captures the overall nonlinear coupling strength, rather than being directly calculated from normalized nonlinear mode-overlap integrals in a full perturbative nonlinear coupled-mode formulation.

We solve Eq. S20 under the slowly varying envelope and rotating-wave approximations, retaining only the resonant coupling terms between the fundamental mode and the third-harmonic mode. Higher-order nonlinear corrections, such as self- and cross-phase modulation terms, are neglected so that the model isolates the effect of PIOT-induced resonance detuning on the THG power dependence. In this effective description, the pump-power dependence enters through the LC rotation angle θ, which determines the fundamental resonance wavelength $\lambda_1(\theta)$. The corresponding fundamental angular frequency is then calculated as $\omega_1(\theta) = 2\pi c/\lambda_1(\theta)$, and the damping rate is defined by $\gamma_1 = \omega_1/2Q_1$. For the third-harmonic mode, we take $\omega_2 = 3\omega_i$, where ω_i is the incident

pump angular frequency, and define $\gamma_2 = \omega_2/2Q_2$.

In the numerical calculation, $Q_1 \approx 40$ is used for the fundamental resonance and $Q_2 \approx 300$ for the third-harmonic resonance, consistent with the linewidths of the corresponding simulated resonances. The effective nonlinear coupling coefficient is fixed at $\kappa = 0.002$ and is taken to be real-valued, so that $\kappa = \kappa^*$. This value keeps the model in the weak-coupling regime, where the third-harmonic modal amplitude remains perturbative compared with the fundamental modal amplitude. In this phenomenological model, κ mainly controls the overall THG magnitude, whereas the upward or downward bending trend of the input-output curve is governed primarily by the detuning trajectory of $\lambda_1(\theta)$ relative to the pump wavelength. The coupled ordinary differential equations are then solved numerically, and the output TH signal is extracted from the calculated third-harmonic modal amplitude.

SI. 8: Linear polarized laser excited mode demonstration in nonlinear regime

In this section, we describe the application of LC rotation by PIOT. As shown in the main text, a large modulation occurs in the FW = 1685 nm with a small power increase from 0 to 30 mW. To clarify this diffraction pattern modulation mechanism, we calculate the evolution of the diffraction pattern with a small step increase in power. According to our best knowledge, the nonlinear diffraction pattern mainly depends on the mode in the nonlinear regime. As the LCs orient from one state to another, the mode will shift to another wavelength, which will change the nonlinear diffraction pattern accordingly. As shown in Fig. S9(A), the 0-order diffraction pattern is enhanced with increasing power, but the 1st order is suppressed in the meantime. Observing the near-field patterns in TH at the $x-y$ plane, Fig. S9(B), the field pattern for 1685 nm TH presents an obvious difference between LC 0 and LC 0.5, indicating that the mode is switched under different rotation states of LC. However, the near field patterns of 1716 nm TH remain constant with LC rotation variation. Therefore, we can confirm that the modulation of the diffraction pattern of TH is caused by mode switching when the LC is oriented.

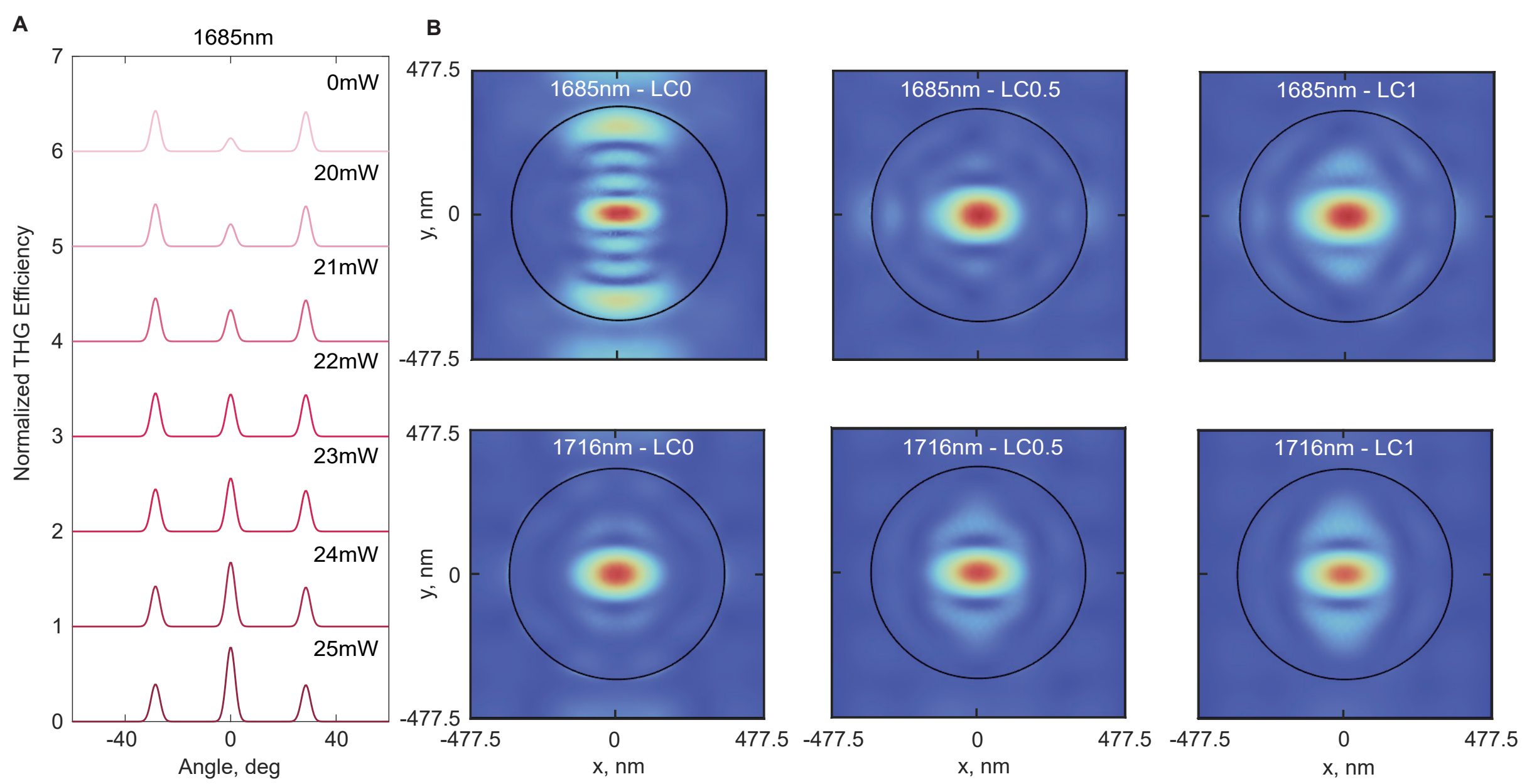

Figure S9: **Nonlinear diffraction order and linear polarized laser excited mode demonstration**

SI. 9: Multipolar decomposition on TH far-field diffraction patterns

This section provides a further analysis and physical interpretation of the third-harmonic (TH) far-field diffraction patterns based on multipolar decomposition. We consider a linearly polarized plane-wave excitation on the magnetic resonance of the metasurface and use the scattering resulting linear electromagnetic field as an external nonlinear current source to calculate the TH response.

For the purpose of multipolar decomposition, the THG is assumed to be emitted into a homogeneous surrounding medium. An effective refractive index is adopted to approximate the LC environment, with $n_{LC} = 1.53$ for the initial state ($LC = 0$) and $n_{LC} = 1.7$ for rotated state $LC = 1$, corresponding to LC director orientations of $\theta = 0$ and $\theta = 90^o$, respectively. The resulting multipolar percentages are demonstrated in Fig. S10.

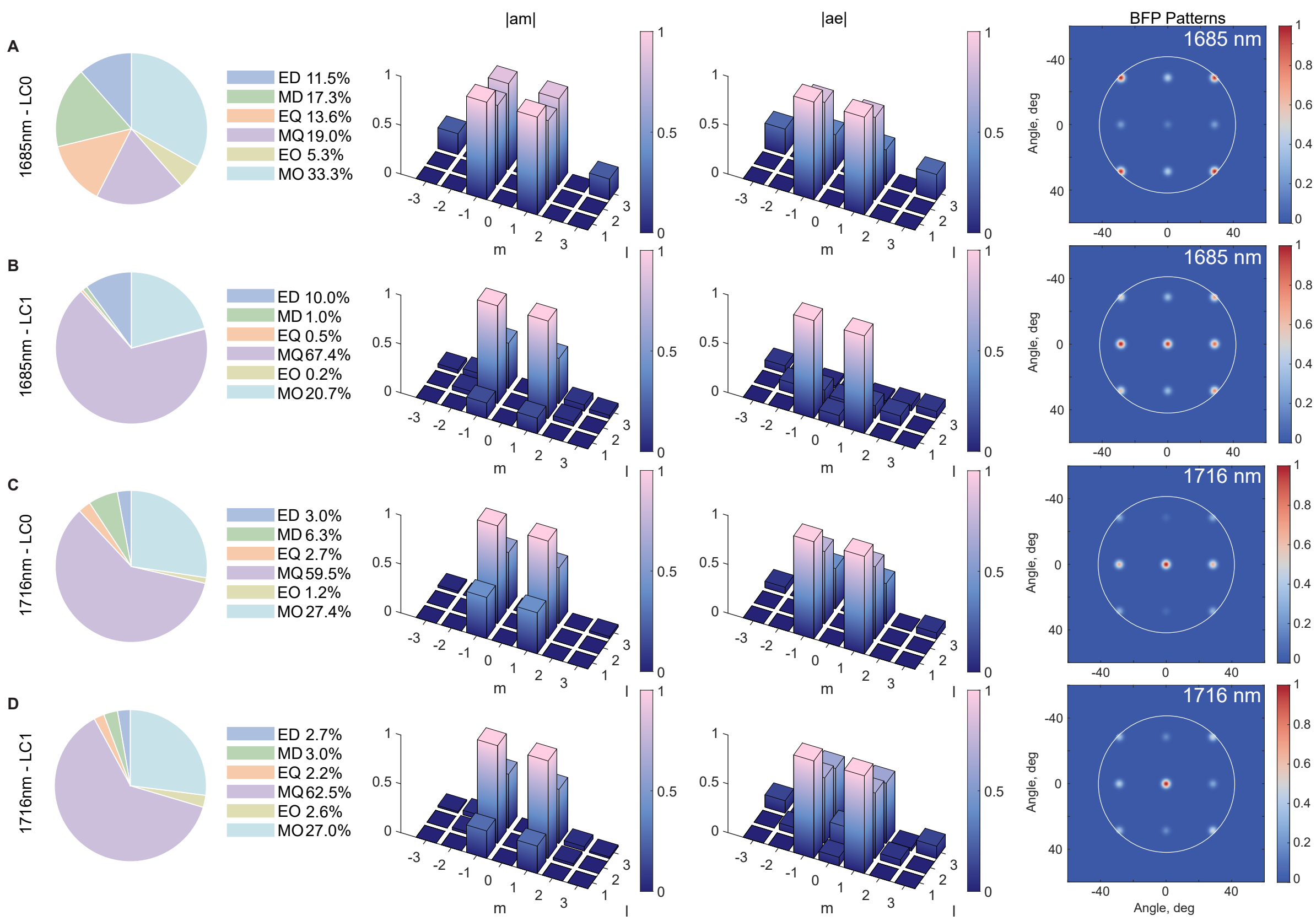

Figure S10: **Multipolar decomposition of TH back focal plane diffraction patterns demonstration**

Fig. S10 presents the multipolar decomposition of the TH field for two representative pump wavelengths, 1685 nm and 1716 nm, each evaluated for both LC states. Panels (A–D) show the

relative contributions of different electric and magnetic multipoles in the form of pie charts. Notably, Fig. S10(A) exhibits a markedly different multipolar composition compared with the other three cases, indicating a substantial redistribution of multipolar contributions induced by the combined effects of excitation wavelength and LC orientation.

It is important to emphasize that the multipolar coefficients ae(l,m) and am(l,m) in the second and third columns describe the angular radiation characteristics of the TH field in a continuous far-field representation. For the periodic metasurface considered here, this continuous angular distribution is subsequently projected onto a discrete set of plane-wave Floquet modes determined by the lattice periodicity. Each Floquet mode corresponds to a specific diffraction order observed in the diffraction pattern image. Consequently, the complex amplitude of a given diffraction order is obtained by evaluating the coherent superposition of all contributing multipolar fields at the corresponding emission direction. The measured intensity of each diffraction order therefore arises from the interference of multiple electric and magnetic multipoles, rather than from a single dominant multipolar component.

SI. 10: Quantification of TH diffraction-order redistribution

To further analyze the modulation of the TH diffraction pattern, we quantify the relative contribution redistribution of each diffraction order at low ($P = 0$) and high ($P = 110\ mW$) pump powers. For each diffraction order (m, n), we define the relative order contribution as

$$C_{m,n}^{\text{low,high}} = \frac{I_{m,n}^{\text{low,high}}}{\sum_{m,n} I_{m,n}^{\text{low,high}}}, \tag{S21}$$

where $I_{m,n}^{\text{low,high}}$ denotes the intensity of the (m, n) diffraction order at low or high pump power, the summation is taken over the nine diffraction orders considered here: $(0, 0)$, $(0, \pm 1)$, $(\pm 1, 0)$, and $(\pm 1, \pm 1)$. The redistribution ratio is then defined as

$$\eta_{m,n} = \frac{C_{m,n}^{\text{high}}}{C_{m,n}^{\text{low}}}. \tag{S22}$$

Here, $\eta_{m,n} > 1$ indicates that the relative contribution of a given diffraction order is enhanced at high pump power, whereas $\eta_{m,n} < 1$ indicates relative suppression.

Table S1: **Relative TH diffraction-order redistribution at FW = 1685 nm.**

Diffraction order (m, n)	I^{low}	I^{high}	$\eta_{m,n}$
$(0, 0)$	0.1380	0.9426	5.9736
$(0, 1)$	0.2359	0.8335	3.0900
$(0, -1)$	0.2306	1.0000	3.7925
$(1, 0)$	0.4251	0.3446	0.7089
$(-1, 0)$	0.4122	0.3374	0.7159
$(1, 1)$	0.9657	0.7233	0.6550
$(1, -1)$	1.0000	0.7607	0.6653
$(-1, 1)$	0.9973	0.5991	0.5254
$(-1, -1)$	0.9731	0.6081	0.5465

Table S1 and Table S2 summarize the calculated redistribution ratios η for FW = 1685 nm and FW = 1716 nm, respectively. At FW = 1685 nm, the relative contribution of the (0,0) TH diffraction order increases by nearly a factor of six, while several higher-order channels are suppressed. At FW

Table S2: **Relative TH diffraction-order redistribution at FW = 1716 nm.**

Diffraction order (m, n)	I^{low}	I^{high}	$\eta_{m,n}$
$(0, 0)$	1.0000	1.0000	0.8900
$(0, 1)$	0.6501	0.2569	0.3516
$(0, -1)$	0.6549	0.4205	0.5715
$(1, 0)$	0.0747	0.1722	2.0518
$(-1, 0)$	0.0748	0.1805	2.1484
$(1, 1)$	0.2955	0.4869	1.4657
$(1, -1)$	0.3098	0.4924	1.4143
$(-1, 1)$	0.3004	0.3746	1.1098
$(-1, -1)$	0.3029	0.3747	1.0994

= 1716 nm, the redistribution is weaker but remains evident, with selected higher-order channels showing approximately two-times enhancement.

These results quantitatively confirm that the PIOT-induced nonlinear modulation is not limited to a small spectral shift, but leads to a substantial redistribution of TH radiation among different diffraction orders. This redistribution reflects the LC-induced change in the TH mode profile, which modifies how the generated nonlinear field couples into different diffraction orders.
